# Microglia-mediated BLA glutamatergic neuronal hyperactivity in the BLA-ACC pathway contributes to stress-induced visceral hypersensitivity and anxiety in rats

**DOI:** 10.1186/s10020-025-01398-w

**Published:** 2025-11-23

**Authors:** Guang-Bing Duan, Jun-Wen Wang, Hui-Hui Sun, Ying Chen, Ewan St John Smith, Ying Huang, Shu-Chang Xu

**Affiliations:** 1 Department of Gastroenterology, Tongji Institute of Digestive Diseases, Tongji Hospital, School of Medicine, Tongji University, Shanghai, China; 2 Department of Pharmacology, University of Cambridge, Cambridgeshire, United Kingdom; 3 Key Laboratory of Spine and Spinal Cord Injury Repair and Regeneration (Ministry of Education), Department of Physiology and Pharmacology, Tongji Hospital, School of Medicine, Tongji University, Shanghai, China

**Keywords:** Microglia, Basolateral amygdala, Anterior cingulate cortex, Visceral hypersensitivity, Anxiety, Neural circuitry, Chemogenetics

## Abstract

**Background:**

Visceral hypersensitivity and anxiety are frequently present as comorbidities in patients with irritable bowel syndrome (IBS). We previously found that both the basolateral amygdala (BLA) and the anterior cingulate cortex (ACC) are involved in stress-related IBS, but the underlying mechanisms remain incompletely understood. In this study, we aimed to determine the role of microglia in the BLA and the BLA-ACC pathway in modulating stress-induced visceral hypersensitivity and anxiety.

**Methods:**

A rat model of chronic stress was established by water avoidance stress (WAS). The abdominal electromyogram was applied to measure visceral sensitivity. The open field test and elevated plus maze test were used to assess anxiety-like behaviors. Minocycline or lipopolysaccharide (LPS) was administrated to inhibit or activate microglia in the BLA. Microglial morphology and neuronal activity were analyzed by immunofluorescence. The expression level of inflammatory mediators in the BLA was determined by quantitative real-time PCR. Chemogenetic approaches were used to manipulate neuronal activity.

**Results:**

WAS induced activation of microglia and secretion of pro-inflammatory mediators in the BLA. Inhibition of microglia in the BLA with minocycline pretreatment prevented the development of visceral hypersensitivity and anxiety-like behaviors induced by WAS. Treatment with LPS could mimic WAS-induced microglia activation in the BLA and result in visceral hypersensitivity and anxiety-like behaviors. Both WAS and LPS significantly enhanced BLA neuronal excitability, whereas inhibition of microglia in the BLA prevented WAS-induced glutamatergic neuron hyperactivity. Inhibition of BLA glutamatergic neurons alleviated both visceral hypersensitivity and anxiety-like behaviors in rats experiencing WAS. Activation of BLA glutamatergic neurons could mimic WAS-induced changes. Selective manipulation of the BLA-ACC glutamatergic pathway bilaterally modulated both visceral hypersensitivity and anxiety-like behaviors. Furthermore, inhibition of BLA microglial activity in WAS rats followed by activation of the BLA-ACC glutamatergic pathway induced visceral hypersensitivity and anxiety-like behaviors.

**Conclusions:**

Overall, our findings reveal that the development of visceral hypersensitivity and anxiety induced by stress is associated with activation of microglia and secretion of pro-inflammatory mediators in the BLA, as well as the hyperactivity of BLA-ACC glutamatergic pathway, thus highlighting the BLA-ACC neurocircuitry as a potential therapeutic target for treating the comorbidities of visceral pain and anxiety.

**Supplementary Information:**

The online version contains supplementary material available at 10.1186/s10020-025-01398-w.

## Background

Irritable bowel syndrome (IBS) is a common disorder of gut-brain interaction, affecting approximately 5% of the world’s population (Black and Ford 2020). Chronic abdominal pain is a hallmark of IBS and is associated with visceral hypersensitivity (Grundy et al. 2019). Triggers of IBS include psychological dysfunction, food, enteric infection and microbial dysbiosis (Ford et al. 2020, 2024; Aguilera-Lizarraga et al. 2022). The global prevalence of mental health conditions has increased in recent decades, and especially in the years following COVID-19 pandemic, an upturn that is likely to cause a dramatic increase in the incidence of IBS (Huang et al. 2019; Collaborators 2021; Marasco et al. 2022). Moreover, the quality of life is much poorer in those IBS patients who also experience a mental health condition (Black and Ford 2020; Grundy et al. 2019). Therefore, the development of effective IBS management strategies necessitates a more profound understanding of the intricate mechanisms that underlie IBS and co-occurring psychological conditions.

Dysregulation of the basolateral amygdala (BLA) excitability is involved in a wide range of conditions such as anxiety disorders and schizophrenia (Janak and Tye 2015; Ma et al. 2024). Accumulating evidence has indicated that the BLA and BLA-associated neural circuitry play significant roles in the regulation of pain-related behaviors (Gao et al. 2023; Valentinova et al. 2023; Tang et al. 2024). There are dense, direct and reciprocal connections between the BLA and the anterior cingulate cortex (ACC), a region widely regarded as a central hub for pain modulation (Fillinger et al. 2017; Bliss et al. 2016). Hyperactivity of BLA neurons projecting to the ACC is critical for the development of chronic pain and depression comorbidity in a neuropathic pain model (Becker et al. 2023). In addition, we previously found that both the BLA and the ACC are involved in stress-induced visceral hypersensitivity and anxiety-like behaviors in rats (Duan et al. 2024; Zhan et al. 2022). However, it is still poorly understood if and how the BLA-ACC pathway is involved in regulating visceral pain and anxiety.

The existence of immune activation within the central nervous system (CNS) has been widely observed in pain or mood disorders, suggesting that the immune system dysfunction may trigger symptoms associated with these conditions (Yuan et al. 2023; Biltz et al. 2022; Parusel et al. 2023). As first-line defense cells of the CNS, microglia become activated rapidly in response to microenvironment challenges (Zhu et al. 2023). Activated microglia can contribute to many conditions with a pivotal mechanism involving the secretion of inflammatory cytokines (Biltz et al. 2022; Inoue and Tsuda 2018). For example, the increased production of interleukin (IL)−1β following spinal cord microglia activation is sufficient to induce chronic somatic pain, whereas inhibition of microglia downregulates the expression of IL-1β and relieves neuropathic pain (Yi et al. 2021a, b). Moreover, nuclear factor kappa-B (NF-κB), tumor necrosis factor-α (TNF-α) and IL-1β produced by activated microglia in the BLA are associated with anxiety- and depressive-like behaviors in mice (Zheng et al. 2021). Notably, these studies highlighted that neuronal hyperactivity is the downstream mechanism of cytokine release, providing further evidence for glia-neuron interactions (Biltz et al. 2022; Inoue and Tsuda 2018). However, the contribution of microglia in the BLA to stress-induced visceral hypersensitivity and anxiety comorbidity has rarely been investigated.

In this study, we aimed to determine the role of microglia in the BLA in modulating psychological stress-indued visceral hypersensitivity and anxiety-like behaviors. Furthermore, we assessed alterations of BLA neural activity in response to microglial activation and inhibition, and investigated whether the BLA-ACC pathway is involved in the comorbidity of visceral pain and anxiety.

## Methods

### Animals

Experiments were conducted using adult male Wistar rats (8–10 weeks, 200–300 g, SLAC ANIMAL Co., Ltd, Shanghai, China). Rats were group-housed with a maximum of four per standard Plexiglas cage and maintained under a 12-hour light/dark cycle (room temperature 24 ± 1 °C), and with food and water available ad libitum. All procedures were approved by the Animal Care and Use Committee of Tongji University School of Medicine (Approval number: TJBC00823201).

### Induction of chronic stress and visceral pain

WAS is a well-established model of chronic psychological stress that induces visceral pain and anxiety (Bradesi et al. 2005; Hong et al. 2015). As described in our previous studies (Duan et al. 2024), rats were placed on a platform (8 cm length × 8 cm width × 10 cm height) mounted in the center of a tank (25 cm length × 25 cm width × 45 cm height), which was filled with fresh water (room temperature 24 ± 1 °C) to 1 cm below the platform top (WAS group) or without water (Ctrl group). Rats underwent this procedure for 1-hour at the same time each day to mitigate against the impact of circadian rhythm variation for ten consecutive days. Body weight was recorded daily as was the number of fecal pellets present in the tank at the conclusion of each WAS procedure.

### Visceromotor response (VMR)

As described previously (Dong et al. 2022; Orock et al. 2024), visceral hypersensitivity (a common indicator of visceral pain) in rodents was revealed by measuring the VMR to colorectal distension (CRD), which was recorded by electromyogram (EMG). Briefly, under general anesthesia (pentobarbital sodium, 50 mg/kg, intraperitoneal, i.p.), the abdominal skin was cut open to expose the left external abdominal oblique muscle into which two sterling silver wires (0.02 cm diameter) were implanted before closing the incision site with sutures. A pre-made flexible latex balloon tied around a medical catheter (0.3 cm diameter) was inserted 4 cm into the colorectal cavity from the rat’s anus and fixed in place with surgical tape around the tail. The rats were allowed to acclimate in a restraint device (2002-A, Yuyan Instruments, Shanghai, China) for 30–45 min before CRD. After a 20-second baseline recording, CRD stimulation (40 or 60 mmHg) by air injection into the inserted balloon was conducted and pressure maintained for 20 s, followed by a 5-minute inter-stimulus interval. Pressure was monitored using a modified sphygmomanometer (Yuwell, Nanjing, China). Each pressure stimulus was repeated three times in the same order (40 and then 60 mmHg) in each rat and VMR was recorded via EMG. The EMG signal was amplified, band-pass filtered between 50 and 500 Hz (Brownlee Precision Model 440, USA) and sampled at 1 kHz (Axon Digidata 1440 A data acquisition system, Molecular Devices, USA), and its traces were analyzed using Clamp Fit 10.7 (Molecular Devices, USA). The difference between the area under the curve (AUC) of the 20-second baseline and 20-second CRD stimulation periods in each rat was analyzed.

### Minocycline or lipopolysaccharide (LPS) treatment

Minocycline treatment Under anesthesia (pentobarbital sodium, 50 mg/kg, i.p.), rats were mounted in a stereotaxic frame (David Kopf Instruments, USA). After shaving and sterilization, a midline cranial skin incision was made to reveal the site for drilling. Two holes were drilled for the placement of two 9.2 mm stainless steel cannulas (24-gauge, RWD Life Science Co., Ltd. China) above the BLA (coordinates: A/P −2.8 mm, L/*R* ± 4.8 mm, D/V −7.2 mm from bregma). Cannulas were fixed to the skull using bone screws and dental cement. Rats were allowed to recover for three days before drug infusion. Reagents were microinfused via a 28-gauge inner cannula connected to a syringe pump (Harvard Apparatus, USA). A total volume of 0.6 µl of either the microglia inhibitor minocycline (20 µg/µl prepared freshly in normal saline (NS); minocycline, HY-17412, MedChemExpress, USA, and NS, G4702, Wuhan Servicebio Technology, China; Mino + WAS group) or NS (NS + WAS group) was microinfused bilaterally into the BLA at a rate of 0.08 µl/min 30 min before the daily WAS procedure. The dose of minocycline was chosen based on previously established effects on microglia inhibition (Zhang et al. 2016a, b).

LPS treatment After baseline VMR assessment, LPS (250 µg/kg prepared in freshly NS; LPS, S7850, Selleck, USA) (LPS group) or NS (NS group) was administered by i.p. injection; this dose of LPS induces a peripheral immune response and increases BLA neuronal activity but does not produce hypotension (Engler et al. 2011). VMR was assessed 2-, 8- and 24-hours after LPS or NS injection. Behavioral assessment was then conducted.

### Assessment of anxiety-like behaviors

Anxiety-like behavior was assessed using the open field test (OFT) and elevated plus maze test (EPMT). Rats were allowed to acclimate to the room where behavioral experiments took place for 24-hours before experiments were conducted. The activity of each rat was monitored using a digital camera placed above the apparatus, which was swabbed with 75% ethanol and dried between tests. The results were analyzed by the EthoVisionXT8.0 Tracking System (Noldus Information Technology, Wageningen, Netherlands).

OFT The OFT was conducted in a black plastic box (80 cm length × 80 cm width × 40 cm height) with rats being allowed to move freely in the box for five minutes. The central zone (CZ) was defined as the 40 cm × 40 cm center area of the box. The total distance traveled, number of rearing events, and the number of entries into the CZ were analyzed.

EPMT The EPMT was performed in an apparatus elevated 70 cm above the ground with two open arms (50 cm length × 10 cm width) and two closed arms (50 cm length × 10 cm width × 40 cm height). After the completion of the OFT, rats were transferred to the EPM and allowed to move freely for five minutes. The frequency of entries into the open arms (OA) and the duration of time spent in the OA were analyzed. An anxiety index was also calculated to evaluate anxiety levels: anxiety index = 1 - [(time spent in the OA/total time on the maze + number of entries into the OA/total entries into the closed and OA)/2].

### Immunofluorescence staining

Brain slice processing and immunofluorescence staining were performed following previously published studies (Duan et al. 2024; Dong et al. 2022). Briefly, 20 μm thick coronal brain slices containing the BLA were sectioned with a cryostat microtome (FS800, RWD Life Science Co., Ltd. China). Slices were permeabilized with phosphate-buffered saline (PBS, pH 7.4) containing 0.2% Triton-X 100 (P0096, Beyotime Biotechnology, China), and then blocked with blocking buffer (P0260, Beyotime Biotechnology, China) for 1 h at room temperature followed by incubation with the appropriate primary antibody: ionized calcium binding adaptor molecule 1 (Iba-1, ab178846, 1:100, Abcam, USA), CaMKIIα (ab52476, 1:200, Abcam, USA) or c-Fos (GB12069, 1:50, Wuhan Servicebio Technology, China) overnight at 4 °C. The sections were then rinsed and incubated with secondary antibodies specific for the primary antibody host: Cy3-labeled goat anti-rabbit IgG (H + L) (A0516, 1:200, Beyotime Biotechnology, China), Alexa Fluor 488-labeled goat anti-rabbit IgG (H + L) (A0423, 1:200, Beyotime Biotechnology, China) or Cy3-labeled goat anti-mouse IgG (H + L) (A0521, 1:200, Beyotime Biotechnology, China) for 2 h. Images of brain slices were captured using a microscope (BX53, Olympus, Japan) or Dragonfly High Speed Confocal Microscope Systems (Dragonfly 200, Oxford Instruments, UK). The same exposure time for each antibody was applied to each slice.

### Image analysis

Six or eight slices from 3 to 4 rats for each group were analyzed by an observer blinded to the experimental design. According to previously published methods (Munshi et al. 2020; Morrison et al. 2017), the number of microglia in bilateral BLA slices was manually counted. A Iba-1 positive cell was considered when the intensity of Iba-1 signal was exceeded two times over the background intensity of negative control slides (without incubation with primary antibody) and had a clearly visible nucleus. The borders of BLA subnuclei were also delineated and subdivided into the lateral amygdala (LA) and basal amygdala (BA) in accordance with a rat brain atlas (Paxinos and Watson 2007). For analysis of microglia morphology, the soma area and processes of microglia were analyzed using ImageJ software (National Institutes of Health, USA) and Excel (Microsoft, USA); de-ramified microglia were defined as those with a soma area > 35 µm^2^ and < 20 processes. For analysis of c-Fos and CaMKIIα co-expression, only cells where the c-Fos signal was observed to be surrounded by the CaMKIIα signal and had a clearly visible nucleus were counted. Total cell number of single CaMKIIα and c-Fos co-expressing cells were counted manually and reported as the ratio of CaMKIIα^+^c-Fos^+^ to total CaMKIIα cells.

### Quantitative real-time PCR (qRT-PCR)

Total RNA was extracted from the bilateral BLA (RNAfast 200, Fastagen, China) and reverse transcribed (RR036A, Takara Bio Inc., China) as previously described (Duan et al. 2024). Quantification was performed as the average of three replicates relative to GAPDH (G3321, Wuhan Servicebio Technology, China). Relative gene expression was analyzed using the △△Ct method. Primers were designed and purchased from Sangon Biotech (Table 1).

**Table 1:** Primer sequences used for qRT-PCR analysis

Gene name	Sequence (5’ ◊ 3’)	
	Forward	Reverse
GAPDH	ACGGCAAGTTCAACGGCACAG	CGACATACTCAGCACCAGCATCAC
IL-1α	CTTTGTGAGTGCTCAGGGAGAAGAC	CTCTGGGAAAGCTGCGGATGTG
IL-1β	ATGCCTCGTGCTGTCTGACC	TTTGTCGTTGCTTGTCTCTCCTTG
IL-2	CACTGACGCTTGTCCTCCTTG	CTCCAGGTGCTGCTGTGTTTC
IL-4	CAACAAGGAACACCACGGAGAAC	CTTCAAGCACGGAGGTACATCAC
IL-6	CTTCCAGCCAGTTGCCTTCTTG	TGGTCTGTTGTGGGTGGTATCC
IL-10	CTGCTATGTTGCCTGCTCTTACTG	GGGTCTGGCTGACTGGGAAG
IL-13	CTCGCTTGCCTTGGTGGTCTTG	TCTGGTCTTGTGTGATGTTGCTCAG
Nlrp3	AGACCTCCAAGACCACGACTG	CCATCCGCAGCCAATGAACAG
Caspase-1	AGTGTAGGGACAATAAATGG	GATGGACCTGACTGAAGC
TNF-α	CACGCTCTTCTGTCTACTGAACTTC	GGGCTACGGGCTTGTCACTC
IFN-γ	ACAACCCACAGATCCAGCACAAAG	CACCGACTCCTTTTCCGCTTCC
M-CSF	CATCATCCTAGTCTTGCTGGCTGTC	AGTTCCACCTGTCTGTCCTCATCC
G-CSF	GGCTCTTCCTCTACCAAGGTCTCC	AGGCAGAAGTGAAGATTGGCATGG
CD11b	GTGCTGGGAGATGTGAATGGAGAC	GGTACTGATGCTGGCTACTGATGC
CD68	GTCTGACCTTGCTGGTACTGCTTG	CGTAGGGCTTGCTGTGCTTCC
Arg-1	AGTGTGGTGCTGGGTGGAGAC	GCGGAGTGTTGATGTCAGTGTGAG

### Retrograde labeling

The procedures for stereotaxic surgery and microinjection were similar to those described above. Briefly, the retrograde tracer rAAV2/Retro-hSyn-mCherry (5.3 × 10^12^ vg/mL, PT-0100, BrainVTA, China) was microinjected bilaterally into the ACC with a total volume of 2.0 µl (coordinates: A/*P* + 1.2 mm, L/*R* ± 0.6 mm, D/V −2.0 mm from bregma). Five weeks after the virus injection, rats were anesthetized, brains collected, and fixed in 4% paraformaldehyde overnight at 4 °C. Coronal slices containing the ACC or BLA (20 μm) were obtained using a cryostat microtome (FS800, RWD Life Science Co., Ltd. China). Localization of mCherry fluorescence was observed using a confocal microscope (Dragonfly 200, Oxford Instruments, UK).

### Chemogenetic stimulation procedures

Manipulation of BLA glutamatergic neuronal activity The activity of glutamatergic neurons in the BLA was manipulated using a chemogenetic strategy. A total volume of 1.6 µl virus encoding inhibitory designer receptors exclusively activated by designer drugs (DREADDs) (rAAV2/9-CaMKIIα-hM4D(Gi)-EGFP, 5.4 × 10^12^ vg/mL, PT-0524, BrainVTA, China) was injected bilaterally into the BLA (coordinates: A/P −2.8 mm, L/*R* ± 4.8 mm, D/V −8 mm from bregma). The virus was microinjected using a 5-µl Hamilton syringe at a rate of 0.08 µl/min. Rats were allowed to recover for four weeks being undergoing the WAS procedure. After establishing WAS, the hM4D(Gi) DREADD was activated, thus inhibiting glutamatergic neurons in the BLA by i.p. injection of clozapine N-oxide (CNO, 2 mg/kg, freshly prepared in NS, S6887, Selleck, USA); NS was injected in control rats. Anxiety-like behaviors were assessed forty-five minutes after the administration of CNO (Gi^+^CNO^+^ group) or NS (Gi^+^CNO^−^ group). A further six rats were microinjected with the same virus in the BLA and were used to assess their VMR in response to CRD before, and forty-five minutes, after CNO administration.

In a separate set of experiments, the role of chemogenetic activation of glutamatergic neurons in the BLA was tested in Ctrl rats. A virus encoding an excitatory DREADD rAAV2/9-CaMKIIα-hM3D(Gq)-EGFP (5.9 × 10^12^ vg/mL, PT-0525, BrainVTA, China) was used in these experiments. Only rats displaying good DREADD expression bilaterally in the BLA were included for further analyses.

Manipulation of the BLA-ACC pathway Similar methods to those described above were used, but with the help of a CRE-DIO system. Specifically, a total volume of 2.0 µl of CRE-virus (rAAV2/Retro-hSyn-CRE-WPRE-hGH, 1.0 × 10^13^ vg/mL, PT-0136, BrainVTA, China) was microinjected bilaterally into the ACC, and then a total volume of 1.6 µl of virus encoding cre-dependent inhibitory or excitatory DREADD (rAAV2/9-CaMKIIα-DIO-hM4D(Gi)-EGFP, 5.5 × 10^12^ vg/mL, PT-3235; or rAAV2/9-CaMKIIα-DIO-hM3D(Gq)-EGFP, 5.8 × 10^12^ vg/mL, PT-1580; BrainVTA, China) was microinjected bilaterally into the BLA. Rats undergoing the WAS procedure were used to analyze the impact of inhibition of the BLA-ACC pathway (Gi). In contrast, Ctrl rats not undergoing WAS were used in experiments examining the impact of activating the BLA-ACC pathway (Gq).

### Statistical analysis

Data analysis was performed using GraphPad Prism (version 9.4.1, GraphPad Software, USA). Data are presented as mean ± standard error of the mean (SEM). Normality was assessed using the Shapiro-Wilk test before analysis. Data analysis between two groups was conducted using a Student’s t test. Data analysis among multiple groups was conducted using a one-way ANOVA followed by either Tukey’s or Dunnett’s post-hoc test. For the analysis of non-normally distributed data, the Mann-Whitney or Kruskal-Wallis test was used. Statistical significance was set at *p* < 0.05. Statistical analysis and sample sizes are provided in the Results and figure legends.

## Results

### Microglia are activated in the BLA of rats experiencing WAS

**Fig. 1:**
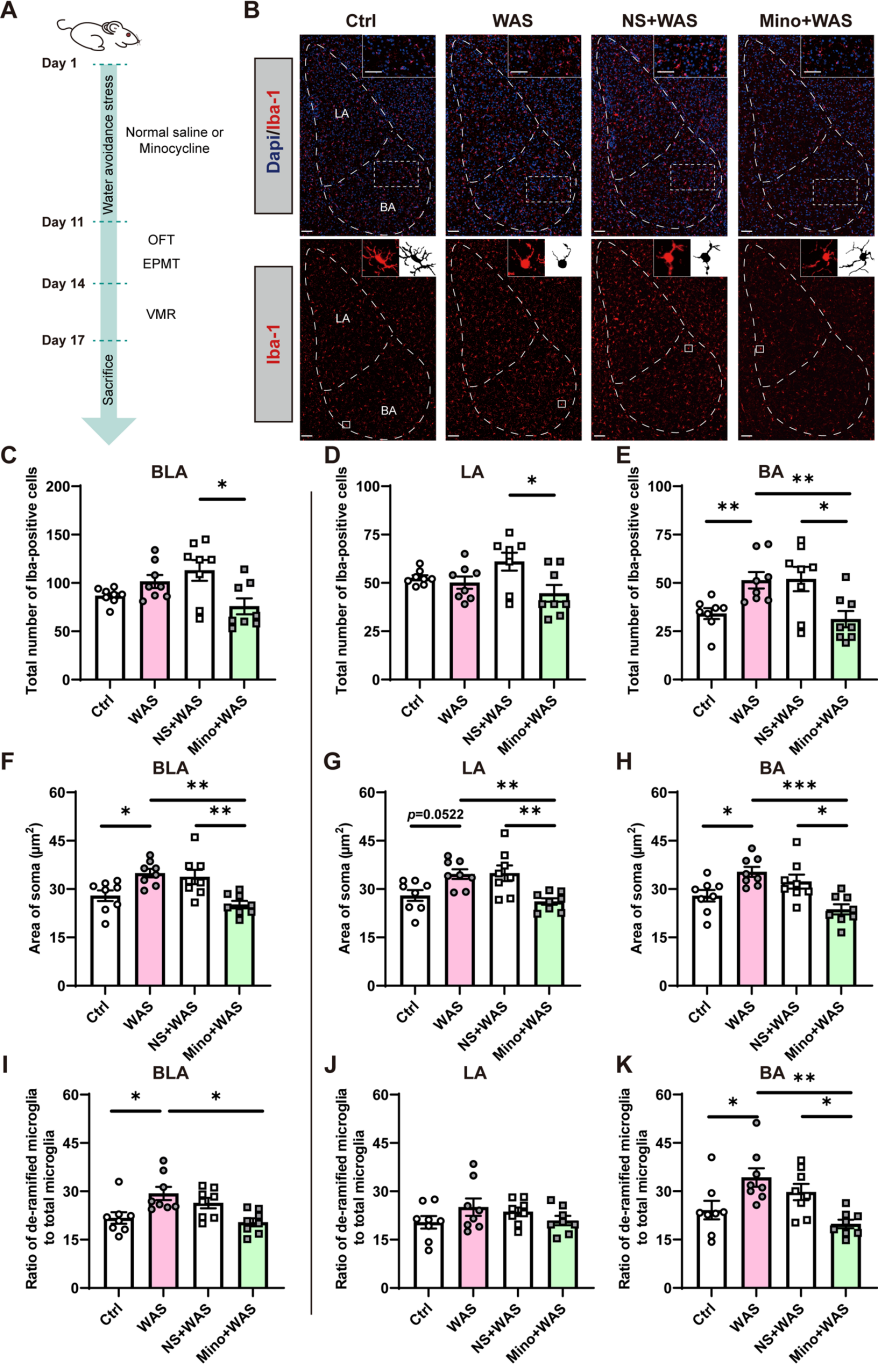
Water avoidance stress (WAS) induces microglial activation in the basolateral amygdala (BLA), which is prevented by minocycline treatment. (**A**) A schematic diagram of the experimental design. OFT, open filed test; EPMT, elevated plus maze test; VMR, visceromotor response. (**B**) Representative images of Iba-1^+^ microglia immunofluorescent staining in the BLA. A/P: −2.4 mm from bregma. Scale bar: 100 μm. Number of Iba-1^+^ microglia in (**C**) BLA, and its sub-nuclei (**D**) lateral amygdala (LA), and (**E**) basal amygdala (BA). Quantification of the microglial soma area in (**F**) BLA, (**G**) LA, and (**H**) BA. Comparison of the ratio of de-ramified microglia/total microglia in (**I**) BLA, (**J**) LA, and (**K**) BA. All data are given as mean ± SEM. *n* = 8 slices from 3–4 rats for each group. *p < 0.05, **p < 0.01, ***p < 0.001, one-­way ANOVA followed by Tukey’s post hoc test or Kruskal–Wallis test

The onset of mood disorders and chronic pain is associated with immune activation in the brain. As the BLA is important in stress-related physiopathology, we first investigated if the alteration of microglial activity in the BLA is crucial to WAS-induced pathological changes. To block the activity of microglia, minocycline was microinjected in the bilateral BLA 30 min before the daily WAS procedure (Fig. 1A). Firstly, Iba-1 immunostaining was performed to count and morphologically characterize microglia in the BLA as previously described (Munshi et al. 2020) (Fig. 1B). The quantified data show that although WAS rats had no significant change in the number of microglia in the whole BLA as well as the LA, a significantly greater number of microglia was observed in the BA of WAS rats compared with Ctrl rats (Fig. 1C-E, *n* = 8 slices from 3 to 4 rats for each group, one-way ANOVA followed by Tukey’s post hoc test, C, F _(3, 28)_ = 4.466, *p* = 0.0110, D, F _(3, 28)_ = 3.568, *p* = 0.0265, E, F _(3, 28)_ = 5.866, *p* = 0.0031). Moreover, WAS rats that had been pretreated with minocycline exhibited a lower number of microglia in the whole BLA, LA, and BA compared with the number of microglia in WAS rats or NS + WAS rats, a difference that was significant for the BA, but only compared to NS + WAS rats for the LA (Fig. 1D, E).

The morphological complexity of microglia was further analyzed using ImageJ. BLA microglia of the WAS group exhibited a significantly larger soma area compared to the Ctrl group, whereas treatment with minocycline prevented the increase in the BLA microglia soma area compared to that of both WAS and NS + WAS rats (Fig. 1F, *n* = 8 slices from 3 to 4 rats for each group, one-way ANOVA followed by Tukey’s post hoc test, F _(3, 28)_ = 8.039, *p* = 0.0005). Similar results were found when the analysis was conducted within the LA and BA subnuclei (Fig. 1G, H, *n* = 8 slices from 3 to 4 rats for each group, one-way ANOVA followed by Tukey’s post hoc test, G, F _(3, 28)_ = 6.788, *p* = 0.0014; H, F _(3, 28)_ = 8.038, *p* = 0.0005).

When taking both soma area and processes into consideration, microglia were divided into ramified and de-ramified groups, the ratio of de-ramified microglia to total microglia being analyzed to further reveal the level of microglial activation. The ratio of de-ramified microglia to total microglia in the BLA as well as BA was significantly increased in WAS rats, but the increase was prevented by inhibiting the microglia activity in Mino + WAS rats (Fig. 1I, K, *n* = 8 slices from 3 to 4 rats for each group, I, Kruskal-Wallis test followed by Dunn’s post hoc test, *p* = 0.0047; K, one-way ANOVA followed by Tukey’s post hoc test, F _(3, 28)_ = 6.419, *p* = 0.0019). No significant difference was found in the ratio of de-ramified microglia to total microglia in the LA among rats in Ctrl, WAS, NS + WAS and Mino + WAS groups (Fig. 1J, *n* = 8 slices from 3 to 4 rats for each group, one-way ANOVA followed by Tukey’s post hoc test, F _(3, 28)_ = 1.320, *p* = 0.2875).

To confirm correct targeting of the BLA, we injected fluorescein 5-isothiocyanate (FITC, a fluorescent tracer) into the BLA through a cannula or a Hamilton micro-syringe, both approaches demonstrating accurate targeting (See Additional File 1: Figure S1). Additionally, the central amygdala (CeA) is close to the BLA, and several well-designed studies have reported the role of microglia in the CeA in stress- or autoimmune-related chronic pain (Yuan et al. 2020, 2021, 2022; Dworsky-Fried et al. 2022). To rule out the potential for off-site effects of minocycline microinjection into the BLA, we analyzed if altered microglial activity occurred in the CeA. The quantified data show that although CeA microglia were activated in WAS rats, minocycline infusions into the BLA had no significant influence on microglial activity in the CeA, which lends further support to accurate targeting of the BLA via our injection technique (See Additional File 1: Figure S2).

Taken together, the results suggest that BLA microglia are activated by chronic psychological stress and that minocycline treatment prevents stress-induced changes.

### Inhibition of microglial activity in the BLA prevents WAS-induced anxiety-like behaviors and visceral hypersensitivity in rats

**Fig. 2:**
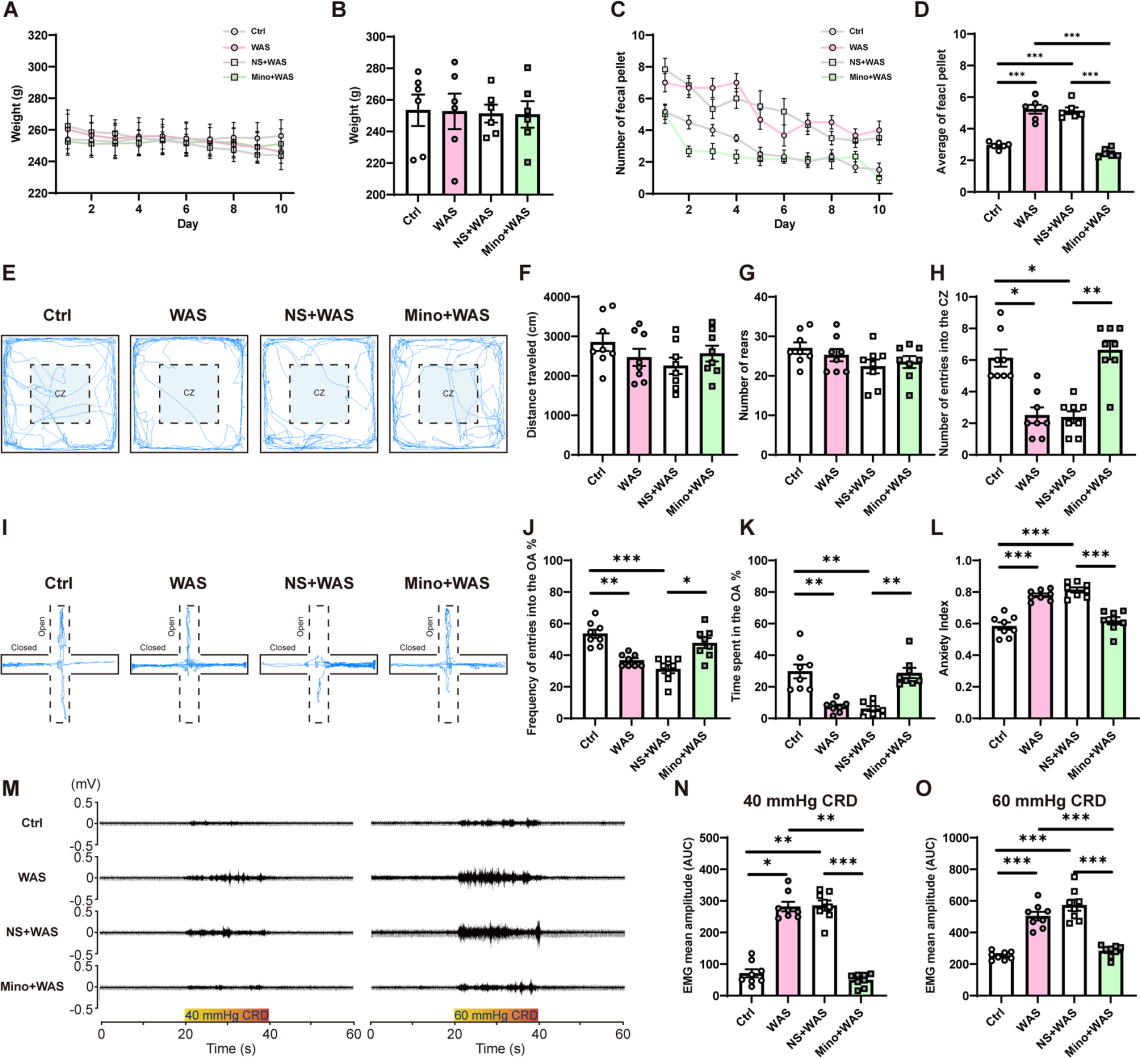
Minocycline treatment in the BLA prevents chronic stress-induced anxiety-like behaviors and visceral hypersensitivity in rats. (**A**) The daily body weight of each group after WAS, and (**B**) the comparison of body weight averaged over ten days. (**C**) The daily number of fecal pellets of each group in the tank after WAS, and (**D**) the comparison of average number of fecal pellets over ten days. n = 6 rats for each group. (**E**) Representative activity traces in the OFT. The comparison of (**F**) total distance traveled, (**G**) total number of rears, and (**H**) number of entries into the central zone (CZ) in the OFT. (**I**) Representative activity traces in the EPMT. The comparison of (**J**) frequency of entries to the open arms (OA), (**K**) time spent in the OA, and (**L**) the anxiety index in the EPMT. (**M**) Representative EMG recordings of VMR amplitude to 40 and 60 mmHg colorectal distension (CRD). Comparison of VMR amplitude to (**N**) 40 and (**O**) 60 mmHg CRD in rats among four groups. All data are given as mean ± SEM. n = 8 rats for each group. *p < 0.05, **p < 0.01, ***p < 0.001, one-way ANOVA followed by Tukey's or Dunnett's post hoc test

To investigate the effects of microglial inhibition in the BLA, anxiety-related behaviors and visceral sensitivity were assessed by several assays. Body weight and fecal pellets were also recorded daily during the WAS procedure. There was no significant influence of WAS or additional minocycline treatment on animal body weight (Fig. 2A, B, *n* = 6 rats for each group, B, one-way ANOVA followed by Tukey’s post hoc test, F _(3, 20)_ = 0.0181, *p* = 0.9966). WAS rats showed an increase in the number of fecal pellets during the daily WAS procedure and increased average fecal pellet output compared to Ctrl rats across the study (Fig. 2C, D). Rats subjected to daily administration of minocycline into the BLA before the WAS procedure exhibited a similar fecal production to Ctrl rats and prevented the increase in the average fecal pellet output compared to WAS and NS + WAS rats (Fig. 2D, *n* = 6 rats for each group, one-way ANOVA followed by Tukey’s post hoc test, F _(3, 20)_ = 58.18, *p* < 0.0001).

OFT and EPMT were conducted to assess anxiety-like behaviors. Representative activity traces in the OFT (Fig. 2E) and quantified data show that there was no significant difference in the total travel distance (Fig. 2F, *n* = 8 rats for each group, one-way ANOVA followed by Tukey’s post hoc test, F _(3, 28)_ = 1.358, *p* = 0.2757), and there was no significant difference in the number of rears among four groups either (Fig. 2G, *n* = 8 rats for each group, one-way ANOVA followed by Tukey’s post hoc test, F _(3, 28)_ = 1.651, *p* = 0.2002). By contrast, WAS significantly decreased the number of entries into the CZ, whereas BLA minocycline treatment prevented the decrease in the number of CZ entries of WAS rats and was no different to the number of CZ entries seen in Ctrl animals (Fig. 2H, *n* = 8 rats for each group, Kruskal-Wallis test followed by Dunn’s post hoc test, *p* < 0.0001). The results suggest that WAS influenced certain anxiety-like behaviors without affecting locomotion and vertical exploration, effects that did not occur in WAS rats treated with minocycline. Representative activity traces in the EPMT (Fig. 2I) and quantified data show that WAS rats made fewer entries into the OA (Fig. 2J, *n* = 8 rats for each group, Kruskal-Wallis test followed by Dunn’s post hoc test, *p* = 0.0001) and spent less time in the OA compared to Ctrl rats (Fig. 2K, *n* = 8 rats for each group, Kruskal-Wallis test followed by Dunn’s post hoc test, *p* < 0.0001). However, whereas NS + WAS rats showed similar behavior to WAS rats, rats in the Mino + WAS group exhibited more entries into and spent more time in the OA compared to NS + WAS rats. Similar changes were found when comparing anxiety indices, WAS and NS + WAS rats showing a higher anxiety index than Ctrl rats, whereas Mino + WAS rats recued the increase in anxiety index compared with rats in NS + WAS group (Fig. 2L, *n* = 8 rats for each group, one-way ANOVA followed by Tukey’s post hoc test, F _(3, 28)_ = 35.88, *p* < 0.0001).

To further evaluate the role of BLA microglia in WAS-induced visceral hypersensitivity, the VMR of animals in response to CRD was assessed as indicated by representative EMG recordings of VMR amplitude to 40 and 60 mmHg CRD (Fig. 2M). The VMR amplitude to 40 or 60 mmHg CRD significantly increased in WAS rats compared to Ctrl rats (Fig. 2N, O). However, minocycline treatment in the BLA prevented the increase in the VMR amplitude induced by WAS compared with that of WAS rats or NS + WAS rats, the Mino + WAS rat group VMR being no different to that of Ctrl rats (Fig. 2N, O, *n* = 8 rats for each group, N: Kruskal-Wallis test followed by Dunn’s post hoc test, *p* < 0.0001; O: one-way ANOVA with Welch’s correction, followed by Dunnett’s post-hoc test, *p* < 0.0001).

Taken together, these data suggest that inhibition of BLA microglial activity prevents WAS-induced anxiety-like behaviors and visceral hypersensitivity.

### Activation of microglia in the BLA with LPS treatment mimics WAS-induced anxiety-like behaviors and visceral hypersensitivity in rats

**Fig. 3:**
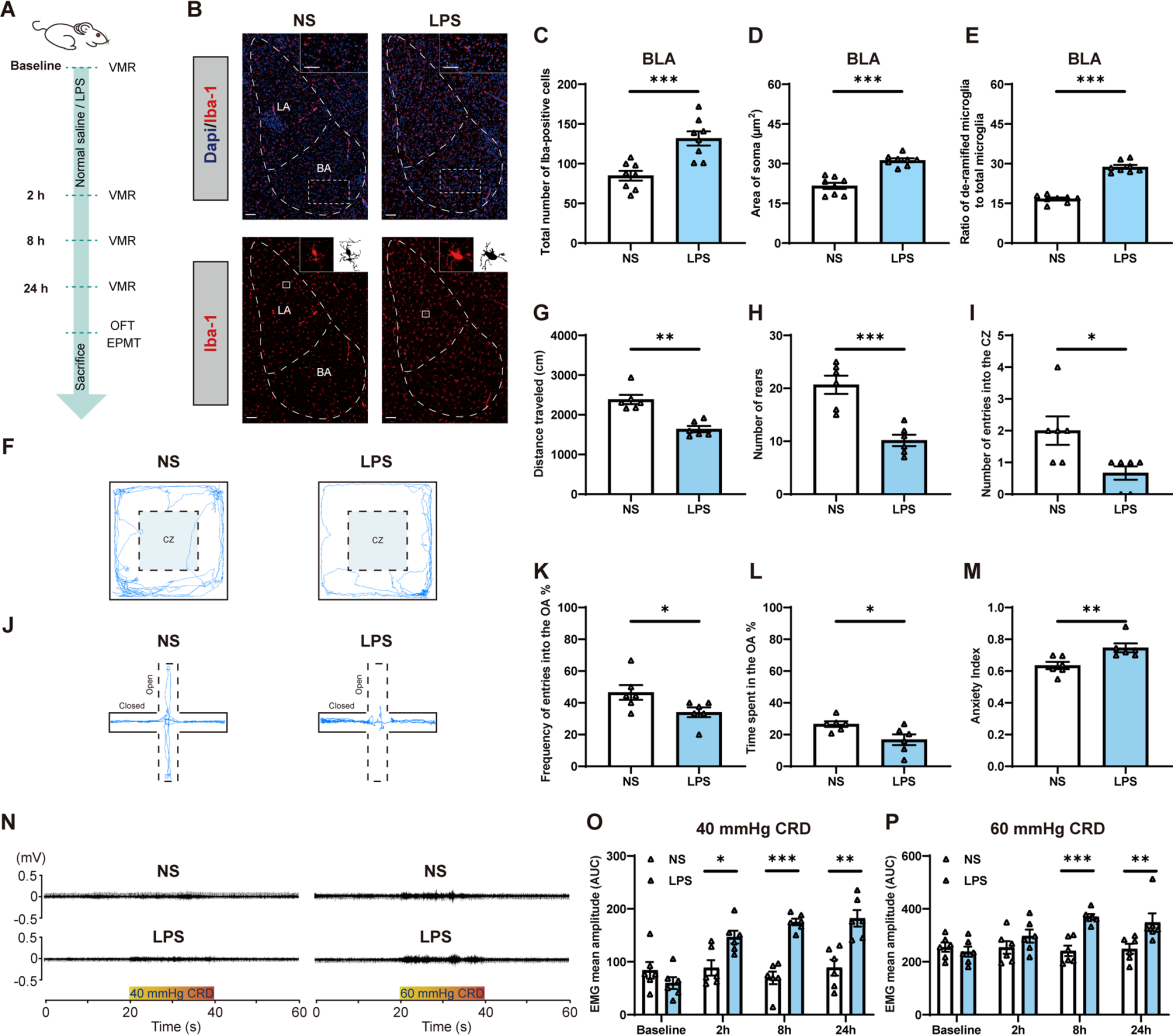
Lipopolysaccharide (LPS) treatment mimics WAS-induced anxiety-like behaviors and visceral hypersensitivity in rats. (**A**) A schematic diagram of the experimental design. (**B**) Representative images of Iba-1^+^ microglia immunofluorescent staining in the BLA 24-hours post LPS or normal saline (NS) treatment. A/P: −2.4 mm from bregma. Scale bar: 100 μm. (**C**) Number of Iba-1^+^ microglia, (**D**) quantification of the microglial soma area, and (**E**) comparison of the ratio of de-ramified microglia/total microglia in the BLA. *n* = 8 slices from 3–4 rats for each group. (**F**) Representative activity traces in the OFT 24-hours post LPS or NS treatment. The comparison of (**G**) total distance traveled, (**H**) total number of rears, and (**I**) number of entries into the CZ in the OFT. (**J**) Representative activity traces in the EPMT. The comparison of (**K**) frequency of entries into the OA, (**L**) time spent in the OA, and (**M**) the anxiety index in the EPMT. (**N**) Representative EMG recordings of VMR amplitude to 40 and 60 mmHg CRD 24-hours post LPS or NS treatment. Comparison of VMR amplitude to (**O**) 40 and (**P**) 60 mmHg CRD in baseline, 2-hours, 8-hours and 24-hours post LPS or NS treatment in rats. All data are given as mean ± SEM. *n* = 6 rats for each group. *p < 0.05, **p < 0.01, ***p < 0.001, two-­tailed unpaired Student’s t­-test or Mann-Whitney test

Administration of LPS was performed to further determine whether activation of microglia in the BLA contributes to anxiety-like behaviors and visceral hypersensitivity (Fig. 3A). The results of Iba-1 immunostaining suggest that LPS injection significantly increased the number of microglia in the BLA (Fig. 3B, C, *n* = 8 slices from 3 to 4 rats for each group, unpaired Student’s t test, *p* = 0.0006). There was a significant increase in soma area in LPS rats (Fig. 3D, *n* = 8 slices from 3 to 4 rats for each group, unpaired Student’s t test, *p* < 0.0001). In addition, LPS rats also exhibited an increased ratio of de-ramified microglia to total microglia in the BLA compared with NS rats (Fig. 3E, *n* = 8 slices from 3 to 4 rats for each group, unpaired Student’s t test, *p* < 0.0001). Similar results were found when the analysis was conducted within the LA and BA subnuclei (See Additional File 1: Figure S3).

Behavioral tests were then conducted. Representative activity traces in the OFT (Fig. 3F) and quantified data show that compared with NS rats, LPS treated rats showed significantly less total travel distance (Fig. 3G, *n* = 6 for each group, Mann-Whitney test, *p* = 0.0022), significantly fewer rears (Fig. 3H, *n* = 6 for each group, unpaired Student’s t test, *p* = 0.0004), and a significantly smaller number of entries into the CZ (Fig. 3I, *n* = 6 for each group, Mann-Whitney test, *p* = 0.0325). Representative EPMT traces (Fig. 3J) and quantified data show that compared with NS rats, LPS treated rats made significantly fewer entries into the OA (Fig. 3K, *n* = 6 rats for each group, unpaired Student’s t test, *p* = 0.0487), spent less time in the OA (Fig. 3L, *n* = 6 rats for each group, unpaired Student’s t test, *p* = 0.0487), and also exhibited a higher anxiety index (Fig. 3M, *n* = 6 for each group, Mann-Whitney test, *p* = 0.0022).

To determine the contribution of LPS to visceral sensitivity in rats, the VMR in response to CRD was measured at baseline, 2-, 8- and 24-hours following i.p. injection of LPS or NS. Representative EMG recordings of the VMR amplitude to 40 and 60 mmHg CRD (Fig. 3N) and quantified data show that while there was no significant difference in baseline visceral sensitivity in LPS or NS groups (Fig. 3O, P, *n* = 6 for each group; O, unpaired Student’s t test, *p* = 0.2337; P, unpaired Student’s t test, *p* = 0.5238), LPS induced a significantly greater VMR amplitude to 40 mmHg, but not 60 mmHg, CRD after 2-hours compared with NS rats (Fig. 3O, P, *n* = 6 for each group; O, Mann-Whitney test, *p* = 0.0411; P, unpaired Student’s t test, *p* = 0.2414). However, CRD of both 40 and 60 mmHg induced a significantly greater VMR amplitude in LPS treated rats 8- and 24-hours after LPS treatment compared with NS rats (Fig. 3O, P, *n* = 6 for each group; 8-hours: O, unpaired Student’s t test, *p* < 0.0001; P, unpaired Student’s t test, *p* = 0.0002; 24-hours: O, unpaired Student’s t test, *p* = 0.0016; P, Mann-Whitney test, *p* = 0.0087).

In addition, a separate experiment was performed to specifically activate BLA microglia by intra-BLA LPS injection. Behavioral tests and visceral sensitivity were measured 24-hours after local NS or LPS injection into the BLA. As expected, similar results were obtained following intra-BLA LPS injection as were achieved following systemic LPS treatment (See Additional File 1: Figure S4).

In summary, these data suggest that activation of microglia with LPS treatment causes both anxiety-like behaviors and visceral hypersensitivity in rats.

### Both chronic psychological stress and LPS treatment alter gene expression of inflammation-associated mediators in the BLA

**Fig. 4:**
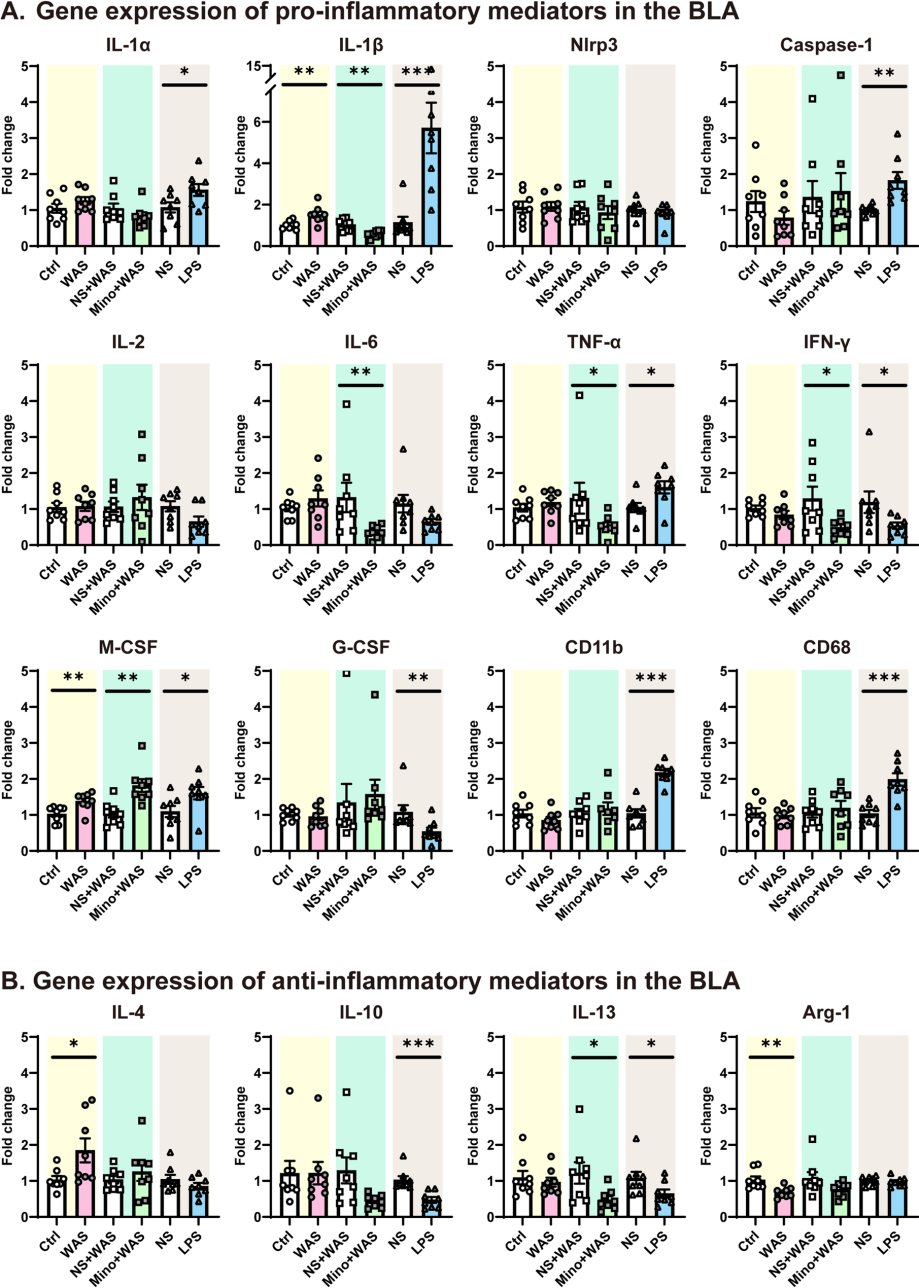
Chronic psychological stress or LPS treatment alters transcriptional profiles of neuroinflammation-related mediators in the BLA, which is prevented by inhibiting microglial activity. (**A**) Fold-changes of different pro-inflammatory cytokines or clusters of differentiation (CD) in the BLA. IL: interleukin, M-CSF: macrophage colony-stimulating factors, G-CSF: granulocyte colony-stimulating factors. (**B**) Fold-changes of different anti-inflammatory cytokines or arginase-1 (Arg-1) in the BLA. All data are given as mean ± SEM. *n* = 8 rats for each group. *p < 0.05, **p < 0.01, ***p < 0.001, Ctrl vs. WAS, NS + WAS vs. Mino + WAS, NS vs. LPS: two-­tailed unpaired Student’s t­-test or Mann-Whitney test

Microglia play a pivotal role in neuroinflammation, their activation resulting in a series of cellular processes including the release of proinflammatory mediators. To determine whether an imbalance in the production of proinflammatory and anti-inflammatory mediators occurred in the BLA in the current study, qRT-PCR was conducted. Firstly, the gene expression of several pro-inflammatory cytokines was tested (Fig. 4A, *n* = 8 rats for each group). The expression of IL-1β was significantly influenced by chronic psychological stress, elevated levels being observed in WAS rats compared with Ctrl rats, an effect not seen in rats treated with minocycline to inhibit microglial activity in the BLA, IL-1β levels being lower in Mino + WAS rats than NS + WAS rats (Fig. 4A, IL-1β, unpaired Student’s t test, Ctrl vs. WAS, *p* = 0.0078; NS + WAS vs. Mino + WAS, *p* = 0.0018). The gene expression of macrophage colony-stimulating factor (M-CSF) also significantly increased in WAS rats, which was maintained after inhibiting microglia activity in WAS rats (Fig. 4A, M-CSF, Mann-Whitney test, Ctrl vs. WAS, *p* = 0.0047; unpaired Student’s t test, NS + WAS vs. Mino + WAS, *p* = 0.0028). A decrease of pro-inflammatory cytokine gene expression was also observed for IL-6, TNF-α and interferon-γ (IFN-γ) in Mino + WAS rats compared with NS + WAS rats although none of these were upregulated by WAS alone (Fig. 4A, IL-6, unpaired Student’s t test with Welch’s correction, Ctrl vs. WAS, *p* = 0.3002, Mann-Whitney test, NS + WAS vs. Mino + WAS, *p* = 0.0047; TNF-α, unpaired Student’s t test, Ctrl vs. WAS, *p* = 0.3478, Mann-Whitney test, NS + WAS vs. Mino + WAS, *p* = 0.0499; IFN-γ, unpaired Student’s t test, Ctrl vs. WAS, *p* = 0.2026, Mann-Whitney test, NS + WAS vs. Mino + WAS, unpaired Student’s t test with Welch’s correction, *p* = 0.0470). No WAS-related changes were observed in the expression of IL-1α, NACHT, LRR, and PYD domains-containing protein 3 (Nlrp3), Caspase-1, IL-2, IL-6, TNF-α, IFN-γ, granulocyte colony-stimulating factor (G-CSF), or the markers of neurotoxic microglia clusters of differentiation (CD) 11b/68 (Fig. 4A, IL-1α, unpaired Student’s t test, *p* = 0.2209; Nlrp3, unpaired Student’s t test, *p* = 0.9233; Caspase-1, unpaired Student’s t test, *p* = 0.2168; IL-2, unpaired Student’s t test, *p* = 0.8802; IL-6, unpaired Student’s t test with Welch’s correction, *p* = 0.3002; TNF-α, unpaired Student’s t test, *p* = 0.3478; IFN-γ, unpaired Student’s t test, *p* = 0.2026; G-CSF, unpaired Student’s t test, *p* = 0.5966; CD11b, unpaired Student’s t test, *p* = 0.2106; CD68, unpaired Student’s t test, *p* = 0.6298).

When examining gene expression changes induced by LPS, pro-inflammatory mediators including IL-1α, IL-1β, Caspase-1, TNF-α, IFN-γ, M-CSF, G-CSF, CD11b, and CD68 were upregulated in the BLA of LPS treated rats compared with levels measured in the BLA of NS rats (Fig. 4A, IL-1α, unpaired Student’s t test, *p* = 0.0411; IL-1β, Mann-Whitney test, *p* = 0.0006; Caspase-1, unpaired Student’s t test with Welch’s correction, *p* = 0.0096; TNF-α, unpaired Student’s t test, *p* = 0.0186; IFN-γ, Mann-Whitney test, *p* = 0.0148; M-CSF, unpaired Student’s t test, *p* = 0.0461; G-CSF, Mann-Whitney test, *p* = 0.0047; CD11b, unpaired Student’s t test, *p* < 0.0001; CD68, unpaired Student’s t test, *p* = 0.0003). However, no significant change was observed in the expression of Nlrp3, IL-2 and IL-6 after LPS treatment (Fig. 4A, Nlrp3, Mann-Whitney test, *p* = 0.5737; IL-2, unpaired Student’s t test, *p* = 0.0528; IL-6, unpaired Student’s t test with Welch’s correction, *p* = 0.0824).

Gene expression of some anti-inflammatory mediators in the BLA was also tested. Compared with Ctrl rats, the expression of IL-4 was increased while expression of arginase-1 (Arg-1) was decreased in WAS rats (Fig. 4B, *n* = 8 rats for each group, IL-4, unpaired Student’s t test, *p* = 0.0346; Arg-1, Mann-Whitney test, *p* = 0.0030). Following treatment with minocycline in WAS rats, the expression of IL-13 was significantly decreased compared with expression in NS + WAS rats (Fig. 4B, IL-13, unpaired Student’s t test with Welch’s correction, *p* = 0.0425). In addition, the expression of IL-10 and IL-13 was down-regulated in rats treated with LPS compared with expression in NS rats (Fig. 4B, IL-10, Mann-Whitney test, *p* = 0.0002; IL-13, Mann-Whitney test, *p* = 0.0490).

Taken together, these data suggest that both chronic stress and LPS disrupt the pro-inflammatory/anti-inflammatory balance in the BLA, treatment with minocycline reversing the increase in pro-inflammatory mediators induced by WAS.

### Chronic stress or LPS treatment activates glutamatergic neurons in the BLA, which is prevented by inhibiting microglial activity

**Fig. 5:**
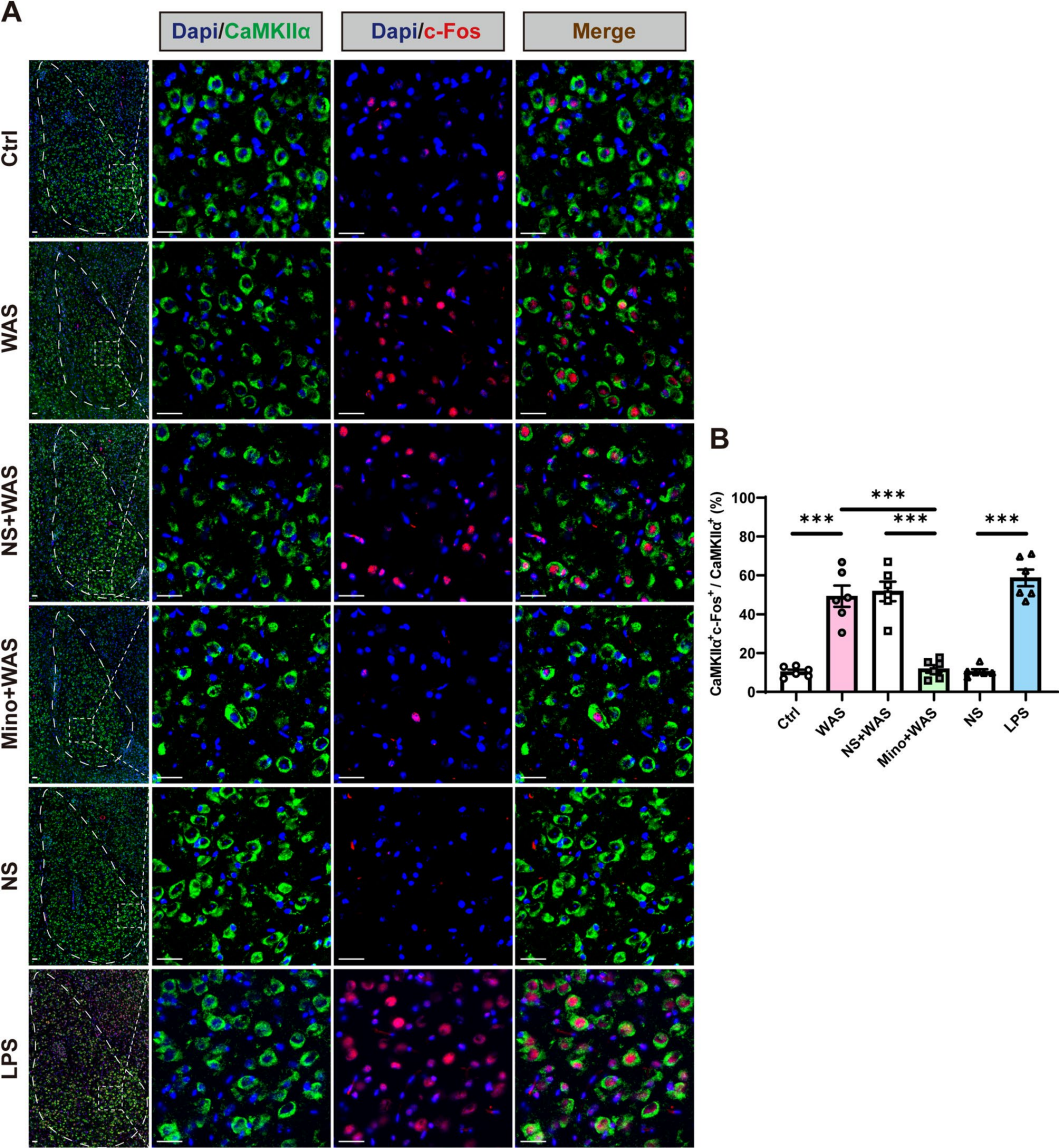
Chronic psychological stress or LPS treatment activates glutamatergic neurons in the BLA, which is prevented by inhibiting microglial activity. (**A**) Representative images of immunofluorescent colocalization staining of CaMKIIα (a marker for glutamatergic neurons) and c-Fos (an immediate-early gene signifying neuronal activation) in the BLA. A/P: −2.4 mm from bregma. Scale bar: 100 μm. (**B**) Quantification of the ratio of CaMKIIα^+^ c-Fos^+^ cells/CaMKIIα^+^ cells in the BLA, indicating the alteration of glutamatergic neuronal activity in the BLA. All data are given as mean ± SEM. *n* = 6 slices from 3 rats for each group. *p < 0.05, **p < 0.01, ***p < 0.001, one­-way ANOVA followed by Tukey’s post hoc test

Interactions between microglia and neurons have been widely investigated in recent years and thus to examine whether the activity of BLA glutamatergic neurons (the predominant neuronal subtype (Sharp 2017) was affected by inhibition or activation of microglia, the co-localization of CaMKIIα (a marker for glutamatergic neurons) and c-Fos (an immediate early gene signifying neuronal activation) was analyzed using immunohistochemistry. Examining the proportion of CaMKIIα/c-Fos double positive cells, it was found that BLA glutamatergic neurons were significantly activated following WAS compared to the level of CaMKIIα/c-Fos double positive cells in the BLA of Ctrl rats. Interestingly, pretreatment with minocycline prevented the activation of glutamatergic neurons, as observed by a significantly decrease in the proportion of CaMKIIα/c-Fos double positive cells in the BLA of Mino + WAS rats compared to WAS or NS + WAS rats (Fig. 5A, B, *n* = 6 slices from 3 rats for each group, one-way ANOVA followed by Tukey’s post hoc test, F _(5, 30)_ = 41.6, *p* < 0.0001). Moreover, glutamatergic neurons were significantly activated following microglia activation with LPS injection compared with levels observed in NS rats (Fig. 5A, B, *n* = 6 slices from 3 rats for each group, one-way ANOVA followed by Tukey’s post hoc test, F _(5, 30)_ = 41.6, *p* < 0.0001).

### Activation of glutamatergic neurons in the BLA contributes to anxiety-like behaviors and visceral hypersensitivity

**Fig. 6:**
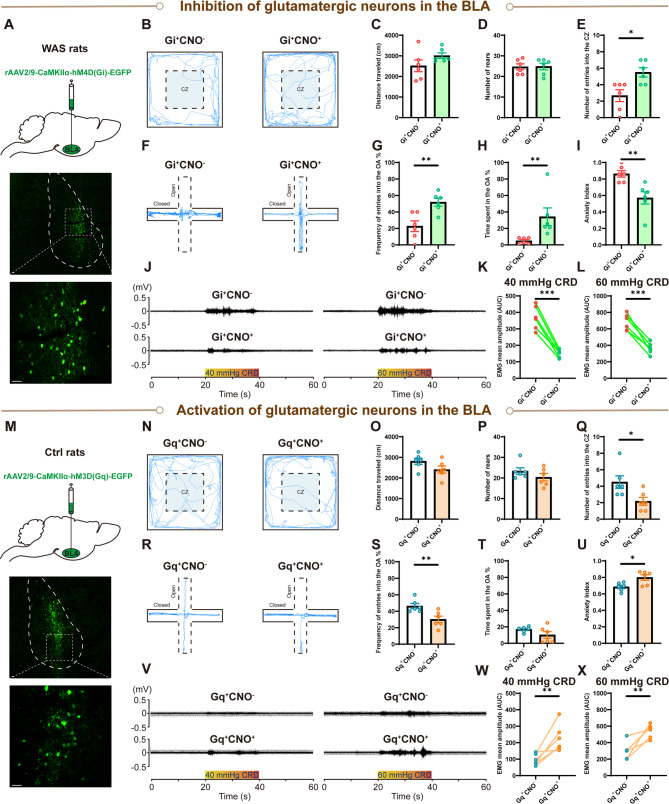
Chemogenetic manipulation of glutamatergic neurons in the BLA modulates anxiety-like behaviors and visceral sensitivity. Inhibition of glutamatergic neurons in the BLA of WAS rats alleviated anxiety-like behaviors and visceral hypersensitivity. (**A**) Graphical representation of Gi-virus microinjection in the BLA and representative images of virus expression in the BLA. A/P: −2.4 mm from bregma. Scale bar: 100 μm. (**B**) Representative activity traces in the OFT. The comparison of (**C**) total distance traveled, (**D**) total number of rears, and (**E**) number of entries into the CZ in the OFT of rats with or without intraperitoneal injection of clozapine N-oxide (CNO), with: Gi^+^CNO^+^, without: Gi^+^CNO^−^. (**F**) Representative activity traces in the EPMT. The comparison of (**G**) frequency of entries to the OA, (**H**) time spent in the OA, and (**I**) the anxiety index in the EPMT. (**J**) Representative EMG recordings of VMR amplitude to 40 and 60 mmHg CRD. Comparison of VMR amplitude to (**K**) 40 and (**L**) 60 mmHg CRD in rats with or without CNO. Activation of glutamatergic neurons in the BLA of normal rats induced anxiety-like behaviors and visceral hypersensitivity. (**M**) Graphical representation of Gq-virus microinjection in the BLA and representative images of virus expression in BLA. A/P: −2.4 mm from bregma. Scale bar: 100 μm. (**N**) Representative activity traces in the OFT. The comparison of (**O**) total distance traveled, (**P**) total number of rears, and (**Q**) number of entries into the CZ in the OFT, with CNO: Gq^+^CNO^+^, without CNO: Gq^+^CNO^−^. (**R**) Representative activity traces in the EPMT. The comparison of (**S**) frequency of entries into the OA, (**T**) time spent in the OA, and (**U**) the anxiety index in the EPMT. (**V**) Representative EMG recordings of VMR amplitude to 40 and 60 mmHg CRD. Comparison of VMR amplitude to (**W**) 40 and (**X**) 60 mmHg CRD with or without CNO. All data are given as mean ± SEM. *n* = 6 rats for each group. *p < 0.05, **p < 0.01, ***p < 0.001, two­-tailed Student’s t­-test or Mann-Whitney test

Since microglia influenced the activity of glutamatergic neurons in the BLA, we further studied the role of glutamatergic neurons using chemogenetic approaches. Firstly, an inhibitory DREADD was delivered to the BLA (Fig. 6A), and representative activity traces in the OFT (Fig. 6B) and quantified data show that compared with rats in the Gi^+^CNO^−^ group, inhibiting activity of glutamatergic neurons in the BLA in WAS rats (Gi^+^CNO^+^ group) did not impact travel distance (Fig. 6C, *n* = 6 for each group, unpaired Student’s t test, *p* = 0.1493), or the number of rears (Fig. 6D, *n* = 6 for each group, unpaired Student’s t test, *p* = 0.9392), but did result in a significantly greater number of entries into the CZ (Fig. 6E, *n* = 6 for each group, unpaired Student’s t test, *p* = 0.0110). In the EPMT, representative activity traces (Fig. 6F) and quantified data show that compared with rats in the Gi^+^CNO^−^ group, rats in the Gi^+^CNO^+^ group made more entries into the OA (Fig. 6G, *n* = 6 rats for each group, unpaired Student’s t test, *p* = 0.0046), spent more time in the OA (Fig. 6H, *n* = 6 rats for each group, Mann-Whitney test, *p* = 0.0022), and exhibited a lower anxiety index (Fig. 6I, *n* = 6 for each group, unpaired Student’s t test, *p* = 0.0059). For visceral sensitivity assessment, representative EMG recordings of VMR amplitude to 40 and 60 mmHg CRD (Fig. 6J) and quantified data show that VMR amplitude was significantly decreased after inhibiting glutamatergic neurons in the BLA at both 40 or 60 mmHg CRD (Fig. 6K, L, *n* = 6 for each group, K, paired Student’s t test, *p* = 0.0002, L, paired Student’s t test, *p* = 0.0004).

Subsequently, experiments using an excitatory DREADD to activate glutamatergic neurons in the BLA of Ctrl rats were conducted to further confirm the role of these neurons in modulating visceral sensitivity and anxiety (Fig. 6M). Representative activity OFT traces (Fig. 6N) and quantified data show that compared with rats in the Gq^+^CNO^−^ group, activating BLA glutamatergic neurons in Ctrl rats (Gq^+^CNO^+^ group) did not impact the distance traveled (Fig. 6O, *n* = 6 for each group, unpaired Student’s t test, *p* = 0.1047) or the number of rears (Fig. 6P, *n* = 6 for each group, Mann-Whitney test, *p* = 0.4048), but did result in a significantly lower number of CZ entries (Fig. 6Q, *n* = 6 for each group, unpaired Student’s t test, *p* = 0.0269). In the EPMT, representative activity traces (Fig. 6R) and quantified data show that compared with rats in the Gq^+^CNO^−^ group, rats in the Gq^+^CNO^+^ group made significantly fewer entries into the OA (Fig. 6S, *n* = 6 rats for each group, unpaired Student’s t test, *p* = 0.0092), spent a similar amount of time in the OA (Fig. 6T, *n* = 6 rats for each group, unpaired Student’s t test with Welch’s correction, *p* = 0.1638), and exhibited a significantly higher anxiety index (Fig. 6U, *n* = 6 for each group, Mann-Whitney test, *p* = 0.0411). Representative EMG recordings of VMR amplitude to 40 and 60 mmHg CRD (Fig. 6V) and quantified data show that regardless of the CRD pressure used, 40 or 60 mmHg, VMR amplitude was significantly increased after activating glutamatergic neurons in the BLA (Fig. 6W, X, *n* = 6 for each group, paired Student’s t tests, *p* = 0.0098 and *p* = 0.0056, respectively).

### The BLA-ACC glutamatergic pathway controls anxiety-like behaviors and visceral hypersensitivity

**Fig. 7:**
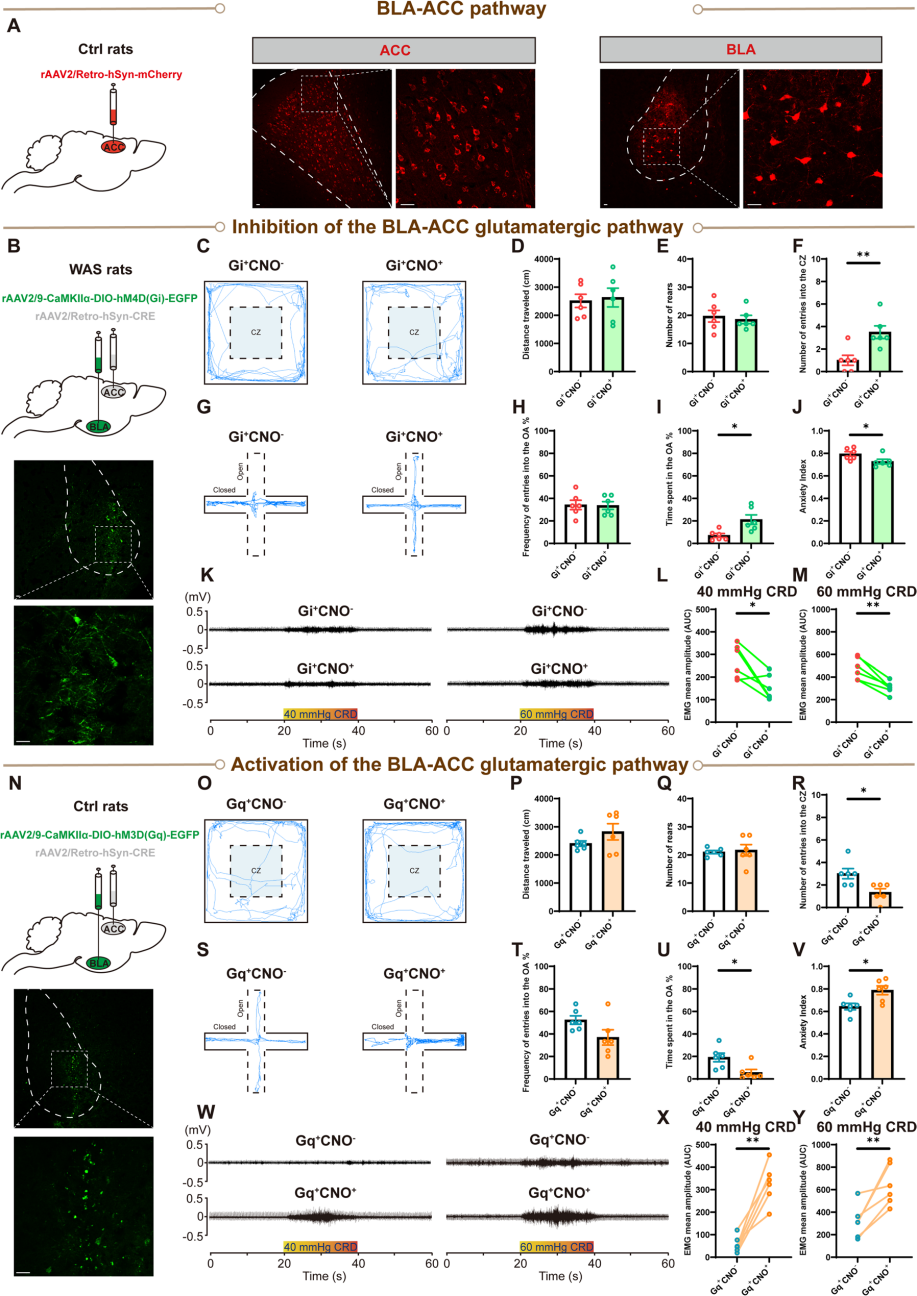
The BLA-anterior cingulate cortex (ACC) pathway controls anxiety-like behaviors and visceral sensitivity. (**A**) The anatomical connectivity between the BLA and the ACC. Graphical representation of retrograde virus microinjection in the ACC (left panel). Representative images of injection site in the ACC (middle panel, A/P: +0.6 mm from bregma) and retrogradely labeled cell bodies in the BLA (right panel, A/P: −2.4 mm from bregma). Scale bar: 100 μm. Inhibition of the BLA-ACC glutamatergic pathway of WAS rats alleviated anxiety-like behaviors and visceral pain. (**B**) Graphical representation of CRE-virus microinjection in the ACC and DIO-Gi-virus microinjection in the BLA, and representative images of virus expression in the BLA. A/P: −2.4 mm from bregma. Scale bar: 100 μm. (**C**) Representative activity traces in the OFT. The comparison of (**D**) total distance traveled, (**E**) total number of rears, and (**F**) number of entries into the CZ in the OFT, with CNO: Gi^+^CNO^+^, without CNO: Gi^+^CNO^−^. (**G**) Representative activity traces in the EPMT. The comparison of (**H**) frequency of entries into the OA, (**I**) time spent in the OA, and (**J**) the anxiety index in the EPMT. (**K**) Representative EMG recordings of VMR amplitude to 40 and 60 mmHg CRD. Comparison of VMR amplitude to (**L**) 40 and (**M**) 60 mmHg CRD in rats with or without i.p. injection of CNO. Activation of the BLA-ACC glutamatergic pathway of Ctrl rats induced anxiety-like behaviors and visceral hypersensitivity. (**N**) Graphical representation of CRE-virus microinjection in the ACC and DIO-Gq-virus microinjection in the BLA, and representative images of virus expression in the BLA. A/P: −2.4 mm from bregma. Scale bar: 100 μm. (**O**) Representative activity traces in the OFT. The comparison of (**P**) total distance traveled, (**Q**) total number of rears, and (**R**) number of entries into the CZ in the OFT, with CNO: Gq^+^CNO^+^, without CNO: Gq^+^CNO^−^. (**S**) Representative activity traces in the EPMT. The comparison of (**T**) frequency of entries into the OA, (**U**) time spent in the OA, and (**V**) the anxiety index in the EPMT. (**W**) Representative EMG recordings of VMR amplitude to 40 and 60 mmHg CRD. Comparison of VMR amplitude to (**X**) 40 and (**Y**) 60 mmHg CRD with or without CNO. All data are given as mean ± SEM. *n* = 6 rats for each group. *p < 0.05, **p < 0.01, ***p < 0.001, two­-tailed Student’s t-­test or Mann-Whitney test

Given that the ACC is considered the center of pain processing and is well-connected with the BLA (Fillinger et al. 2017; Bliss et al. 2016), we hypothesize that the BLA may work together with ACC to cause visceral hypersensitivity following activation of BLA glutamatergic neurons. Firstly, to confirm the anatomical connectivity between the BLA and the ACC, the retrograde labeling virus rAAV/Retro-hSyn-mCherry was microinjected into the ACC, and brain slices containing the BLA were collected five weeks post- injection. Consistent with previous analysis (Becker et al. 2023), we observed densely labeled cell bodies in the BLA following injection of the retrograde labeling virus into the ACC (Fig. 7A).

CRE-DIO systems were next combined with chemogenetic tools to elucidate the role of the BLA-ACC pathway in the WAS model (Fig. 7B). Representative activity OFT traces (Fig. 7C) and quantified data show that inhibition of the BLA-ACC pathway in WAS rats (Gi^+^CNO^+^ group) resulted in a similar travel distance (Fig. 7D, *n* = 6 for each group, unpaired Student’s t test, *p* = 0.7779) and number of rears (Fig. 7E, *n* = 6 for each group, unpaired Student’s t test, *p* = 0.6536) compared to rats in the Gi^+^CNO^−^ group, but inhibition of the BLA-ACC pathway in WAS rats resulted in significantly more entries into the CZ (Fig. 7F, *n* = 6 for each group, unpaired Student’s t test, *p* = 0.0059). In the EPMT, representative activity traces (Fig. 7G) and quantified data show that compared with rats in the Gi^+^CNO^−^ group, rats in the Gi^+^CNO^+^ group made a similar number of entries into the OA (Fig. 7H, *n* = 6 rats for each group, unpaired Student’s t test, *p* = 0.9174), but spent significantly more time in the OA (Fig. 7I, *n* = 6 rats for each group, Mann-Whitney test, *p* = 0.0117), and exhibited a significantly lower anxiety index (Fig. 7J, *n* = 6 for each group, unpaired Student’s t test, *p* = 0.0436). For visceral sensitivity assessment, representative EMG recordings of VMR amplitude to 40 and 60 mmHg CRD (Fig. 7K) and quantified data show that VMR amplitude was significantly decreased after inhibiting the BLA-ACC pathway at both 40 and 60 mmHg CRD (Fig. 7L, M, *n* = 6 for each group, paired Student’s t tests, *p* = 0.0233 and *p* = 0.0019, respectively).

Experiments enabling selective activation of the BLA-ACC glutamatergic pathway were next conducted in Ctrl rats (Fig. 7N). Representative activity traces of the OFT (Fig. 7O) and quantified data show that compared to rats in the Gq^+^CNO^−^ group, rats in the Gq^+^CNO^+^ group showed a similar travel distance (Fig. 7P, *n* = 6 for each group, unpaired Student’s t test, *p* = 0.1981) and number of rears (Fig. 7Q, *n* = 6 for each group, unpaired Student’s t test, *p* = 0.7611), but undertook significantly fewer entries into the CZ (Fig. 7R, *n* = 6 for each group, unpaired Student’s t test, *p* = 0.0136). In the EPMT, representative activity traces (Fig. 7S) and quantified data show that compared with rats in the Gq^+^CNO^−^ group, rats in the Gq^+^CNO^+^ group had a lower tendency to enter into the OA (Fig. 7T, *n* = 6 rats for each group, unpaired Student’s t test, *p* = 0.0739), spending significantly less time in the OA (Fig. 7U, *n* = 6 rats for each group, Mann-Whitney test, *p* = 0.0152), and exhibited a significantly higher anxiety index (Fig. 7V, *n* = 6 for each group, unpaired Student’s t test, *p* = 0.0140). Representative EMG recordings of VMR amplitude to 40 and 60 mmHg CRD (Fig. 7W) and quantified data show that regardless of CRD pressure, VMR amplitude was significantly increased after activating the BLA-ACC pathway (Fig. 7X and Y, *n* = 6 for each group, paired Student’s t test, *p* = 0.0011 and *p* = 0.0091, respectively).

Furthermore, to confirm that the activity alteration of the BLA-ACC pathway is downstream of BLA microglial activity, we firstly inhibited BLA microglial activity in WAS rats and then activated the BLA-ACC pathway using chemogenetic approaches. The data suggest that inhibition of BLA microglial activity in WAS rats followed by activation of the BLA-ACC glutamatergic pathway is sufficient to induce anxiety-like behaviors and visceral hypersensitivity (See Additional File 1: Figure S5).

## Discussion

**Fig. 8:**
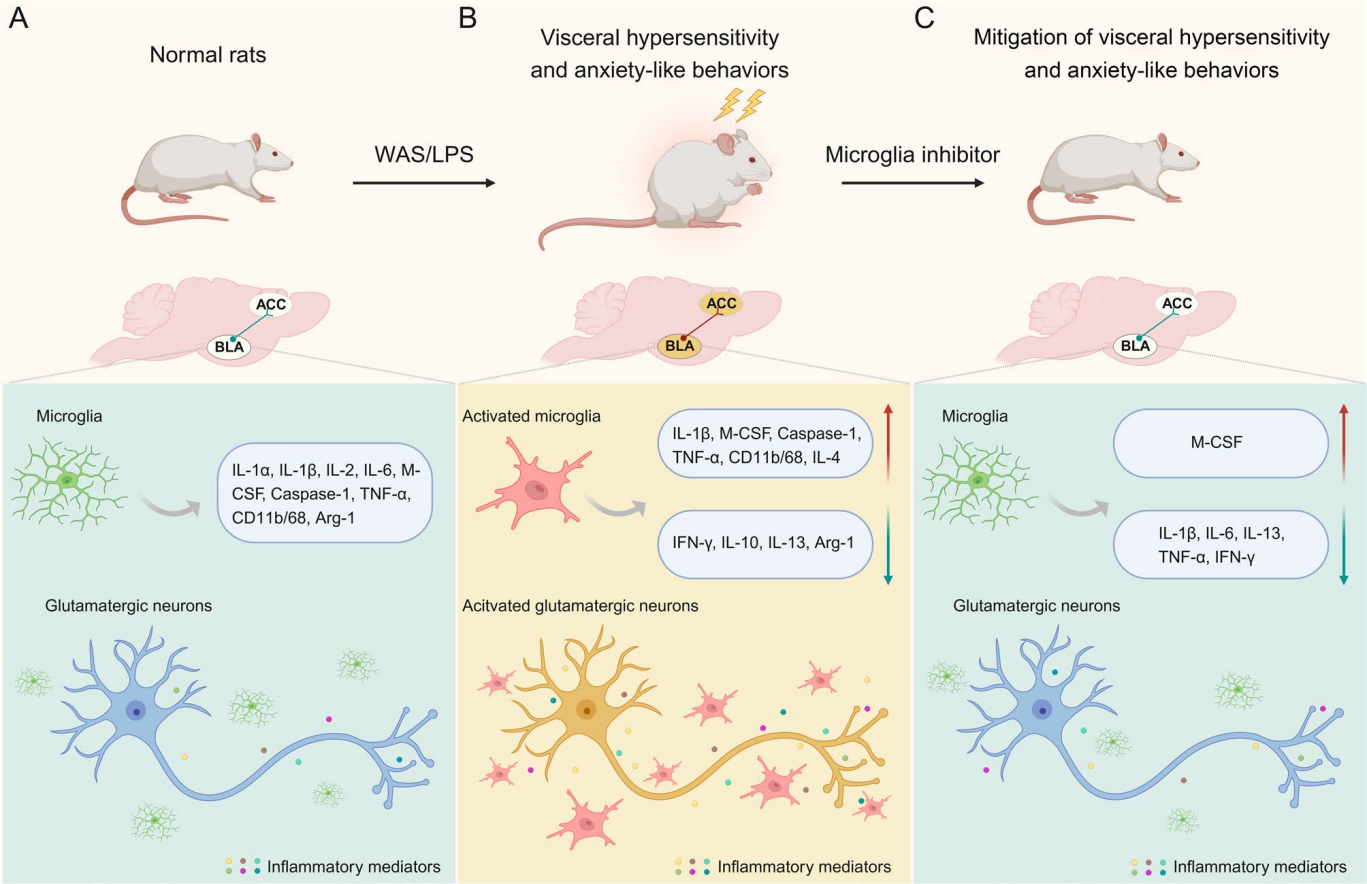
A graphical abstract shows that minocycline-induced microglia inhibition in the BLA induces analgesic and anxiolytic effects by modulating glutamatergic neuronal activity in rats. (**A**) Homeostasis in normal rats. (**B**) The onset of visceral hypersensitivity and anxiety-like behaviors induced by WAS or LPS treatment is associated with activation of microglia and secretion of pro-inflammatory mediators in the BLA. Both WAS and LPS significantly enhance BLA neuronal excitability. Moreover, activation of the BLA-ACC glutamatergic pathway induces visceral hypersensitivity and anxiety-like behaviors. (**C**) Pretreatment with microglia inhibitor (minocycline) in the BLA inhibits WAS-induced microglia activation, production of inflammatory mediators and glutamatergic neuron activation, thereby preventing the development of both visceral hypersensitivity and anxiety-like behaviors in rats. Selective inhibition of the BLA-ACC glutamatergic pathway of WAS rats alleviates visceral hypersensitivity and anxiety-like behaviors. Created with BioRender.com

Chronic abdominal pain and anxiety are highly prevalent in individuals with IBS, but the development of treatments for this comorbidity is plagued by unclear mechanisms underpinning the symptoms presented (Grundy et al. 2019; Zamani et al. 2019). Here, we explored the role of BLA microglia and the BLA-ACC neural circuitry in the pathogenesis of visceral hypersensitivity and anxiety-like behaviors in rats induced by chronic stress using the WAS model. We observed that targeted minocycline-induced microglia inhibition in the BLA produced analgesic and anxiolytic effects in the WAS model, effects recapitulated by inhibiting BLA glutamatergic neurons or inhibiting the BLA-ACC pathway in WAS rats, whereas activating BLA glutamatergic neurons or exciting the BLA-ACC pathway in Ctrl rats induced visceral hypersensitivity and anxiety-like behaviors (Fig. 8).

Due to its low molecular weight (495 KDa) and lipid solubility, minocycline can easily cross the blood-brain barrier and accumulate in the CNS (Romero-Miguel et al. 2021). Beyond its well-established broad-spectrum antibiotic properties, minocycline can also inhibit microglia activation and proliferation, thereby exerting anti-inflammatory, anti-oxidant and neuro-protective effects within the CNS (Hellmann-Regen et al. 2022; Xue and Yong 2020). However, systemic minocycline administration is not microglia-specific, also affecting other immune cells including neutrophils and macrophages (Moller et al. 2016). Therefore, to limit its peripheral effects, we used cannulation and intra-BLA microinjection of minocycline (Duan et al. 2024; Dong et al. 2022). We found that WAS induced anxiety-like behaviors and visceral hypersensitivity in rats. Moreover, WAS rats that were treated with minocycline showed greater exploratory behavior (more entries into the CZ in the OFT) and exhibited a lower anxiety index in the EPMT compared with WAS rats receiving only saline. In addition to the anxiolytic role of minocycline, previous studies reported that it could improve pain-related behavior in animal models of visceral pain induced by CRD (Zhang et al. 2016a, b). Similarly, our VMR data showed that minocycline pretreatment significantly alleviated WAS-induced visceral hypersensitivity, thus indicating the potent analgesic effect of minocycline. Given these findings, minocycline, or more specifically targeting microglia within the BLA, may be a promising therapeutic approach for treating the comorbidity of anxiety and chronic abdominal pain that often occurs in IBS. However, minocycline is a non-selective microglia inhibitor with several mechanisms involved that mediate its beneficial effects, and thus, despite the route of administration chosen, it would be important to compare the effects of minocycline and microglia-specific inhibitors such as PLX3397 in future studies.

LPS is a major structural component of the outer membrane in Gram-negative bacteria, which has been widely recognized as a reliable tool for activating microglia in neuroinflammation research (Zheng et al. 2021; Huang et al. 2020). Here, we showed that measurements for exploration, locomotor activity and anxiety in both the OFT and the EPMT were significantly affected 24-hours after LPS administration, and these results were in line with previous studies (Engler et al. 2011; Munshi and Rosenkranz 2018). Moreover, following i.p. administration of LPS, we found that visceral hypersensitivity developed within 2–8 h and was maintained for at least 24 h compared to rats with NS treatment, thus indicating that the LPS-induced acute inflammatory response can trigger visceral pain. Findings from a series of randomized controlled trials have also demonstrated that LPS-treated subjects showed increased visceral sensitivity reflected by decreased pain thresholds and altered pain ratings compared to saline controls, functional magnetic resonance imaging further highlight the role of altered central processing of visceral pain stimuli in LPS-induced visceral hypersensitivity (Benson et al. 2012, 2015; Wegner et al. 2015).

Numerous studies have revealed that microglia activation induced by stress is highly related to the development of pain and anxiety (Biltz et al. 2022; Hoffmann and Beyer 2020). Since we have observed the effects of minocycline and LPS on behaviors and visceral sensitivity in rats, morphological changes of microglia that reflect the microglia activation status in the BLA were also investigated. Consistent with a previous study that focused on the role of microglia in the BLA in a repeated social defeat stress model (Munshi et al. 2020), we found that WAS significantly increased the number of Iba-1^+^ microglia in the BA nuclei but not in the LA nuclei. Morphological remodeling of microglia also occurred following chronic WAS, characterized by an increase in the soma area and a decrease in the number of processes. Notably, pretreatment with minocycline in the BLA prevented these WAS-induced morphological alterations in microglia. In addition, systemic administration of LPS induced a significant increase in the number of microglia and soma area, along with a decrease in the number of microglia processes in the BLA. These findings suggest that the development of comorbid visceral hypersensitivity and anxiety-like behaviors is associated with microglia activation in the BLA, and that minocycline may exert its analgesic and anxiolytic effects by inhibiting microglia activation in the BLA.

It has been shown that activated microglia can release inflammatory cytokines that propagate immune signals within the brain, which are involved in the pathogenesis of pain and mental disorders (Biltz et al. 2022; Inoue and Tsuda 2018). For instance, chronic mild stress significantly increases the expression of both the Nlrp3 inflammasome and IL-1β in the hippocampus, resulting in depressive- and anxiety-like behaviors in rats (Wang et al. 2018). Additionally, once released, IL-1β can directly act on neurons within the dorsal horn and enhance excitatory neurotransmission, closely associated with central sensitization and pain hypersensitivity (Kawasaki et al. 2008). Moreover, IL-1β inhibitors have proven beneficial and safe for patients with pain conditions such as gout and intervertebral disc degeneration (Schlesinger et al. 2023; Wang et al. 2020). Here, we found that among pro-inflammatory mediators, chronic stress only markedly increased the expression of IL-1β and M-CSF in the BLA, treatment with minocycline preventing the elevation in IL-1β, but failing to down-regulate the WAS-induced increase in M-CSF expression. We also observed the most significant change in gene expression for IL-1β in LPS-treated rats. These results suggest that both WAS and LPS may promote neuroinflammation by activating the IL-1β signal pathway in the BLA.

Converging lines of evidence have revealed that activated microglia can affect neuronal activity directly via membrane-membrane communication and indirectly through microglia-derived mediators such as proinflammatory cytokines and neurotrophic factors (Cserep et al. 2021; Basso et al. 2017). Following microglia activation, presynaptic glutamate release is enhanced, thus leading to disruption of excitatory/inhibitory balance and enhanced intrinsic excitability in BLA neurons, changes that can be mitigated by blockade of microglia activation (Zheng et al. 2021; Munshi et al. 2020; Wang et al. 2024). Furthermore, LPS injection also increases the activity of BLA neurons 2 h post-treatment (Engler et al. 2011). In our study, we investigated the activity change of glutamatergic neurons, the predominant neuronal type in the BLA, by immunohistochemistry. The results showed that a greater number of BLA glutamatergic neurons were activated following WAS or LPS treatment compared to controls, and that pretreatment with minocycline in the BLA could prevent the WAS-induced enhancement of glutamatergic activity. Subsequently, by employing chemogenetic techniques, we showed that inhibition of glutamatergic neurons in the BLA could significantly relieve anxiety-like behaviors and visceral hypersensitivity in rats subjected to WAS. In contrast, activation of BLA glutamatergic neurons in Ctrl rats could mimic the stress-induced symptoms observed in rats undergoing WAS. Therefore, these results demonstrate that the hyperactivity of glutamatergic neurons in the BLA induced by chronic stress is crucial to the modulation of both anxiety and pain.

The viral retro-grade tracing results in this study confirmed the density of neurons in the BLA that project to the ACC, as has been observed in previous studies which established that these ACC projecting neurons are glutamatergic (Becker et al. 2023; Chang et al. 2024). Although the functions of the BLA and the ACC in pain and mood regulation are well-known, whether the BLA-ACC pathway is a critical neuro-circuitry for visceral pain is poorly understood (Chang et al. 2024). To address this gap, we manipulated the neuronal activity of BLA glutamatergic neurons which had projections to the ACC using the CRE/DIO-based chemogenetic strategy in WAS or Ctrl rats. Our results for the first time uncovered that inhibition of the BLA-ACC pathway blocked the occurrence of WAS-induced visceral hypersensitivity and anxiety-like behaviors. To further confirm the importance of this pathway, activation of BLA-ACC projection neurons in Ctrl rats was seen to be sufficient to trigger the visceral hypersensitivity and anxiety-like behaviors that occurred in rats undergoing WAS. These results suggest that microglia activation in the BLA may cause visceral hypersensitivity and anxiety by activating the specific BLA-ACC circuitry.

Despite the importance of our findings, there are some limitations in the present study. First, experiments were only performed on male rats to avoid the possible effects of estrogenic hormones on pain perception, which perhaps obscures the potential sex differences in stress-induced behaviors. Second, asymmetries in the left and right BLA are probably associated with stress and emotion processing (Valipour et al. 2024a, b; Guadagno et al. 2020; Ocklenburg et al. 2022), and in this study both sides of the BLA were analyzed together without side-specific analysis. Finally, the design of the control group in our chemogenetic experiments included only saline injection but did not involve DREADD-free animals. Although previous evidence has shown that CNO treatment with low doses (2 mg/kg) had no significant influence on pain- and anxiety-like behaviors in DREADD-free animals (Dong et al. 2022; Xie et al. 2023; Wang et al. 2023; Qi et al. 2022), more reliable DREADD agonists such as compound 21 and deschloroclozapine could be applied in future studies to eliminate potential off-target effects of CNO (Chakrabarti et al. 2020; Nagai et al. 2020).

## Conclusions

In conclusion, our work demonstrated that the development of visceral hypersensitivity and anxiety-like behaviors was mediated by microglial activation in the BLA, followed by the increased secretion of pro-inflammatory mediators and hyperactivity of BLA-ACC glutamatergic neurocircuitry. We also provided new evidence for the beneficial effects of attenuating neuroinflammation on stress-induced comorbidity of chronic pain and anxiety.

## Supplementary Information

Supplementary Material 1.

## Data Availability

No datasets were generated or analysed during the current study.

## Abbreviations

IBS: Irritable bowel syndrome
BLA: Basolateral amygdala
ACC: Anterior cingulate cortex
WAS: Water avoidance stress
VMR: Visceromotor response
LPS: Lipopolysaccharide
OFT: Open field test
EPMT: Elevated plus maze test
Iba-1: Ionized calcium binding adaptor molecule 1
LA: Lateral amygdala
BA: Basal amygdala
CeA: Central amygdala
