# Supplementary material for: Microglia-mediated BLA glutamatergic neuronal hyperactivity in the BLA-ACC pathway contributes to stress-induced visceral hypersensitivity and anxiety in rats
Source: Mol Med. 2025 Nov 23;31:343. doi: 10.1186/s10020-025-01398-w (PMC12751297; doi:10.1186/s10020-025-01398-w)
Supplement: Supplementary file 1 — Supplementary Material 1. [file 10020_2025_1398_MOESM1_ESM.docx]

**Additional File 1**

**
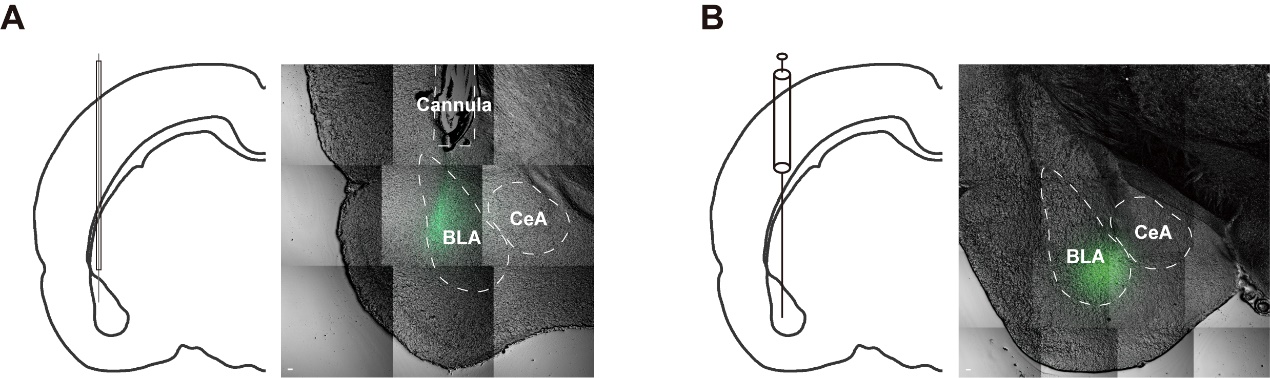
Figure S1**

**Figure S1** Representative histological images showing the injection of fluorescein 5-isothiocyanate (FITC) into the basolateral amygdala (BLA) through **(A)** a cannula (0.6 μl) or **(B)** a Hamilton micro-syringe (0.8 μl). Rats were sacrificed 3-hours after the injection. A/P: -2.4 mm from bregma. Scale bar: 100 μm. CeA: central amygdala.

**
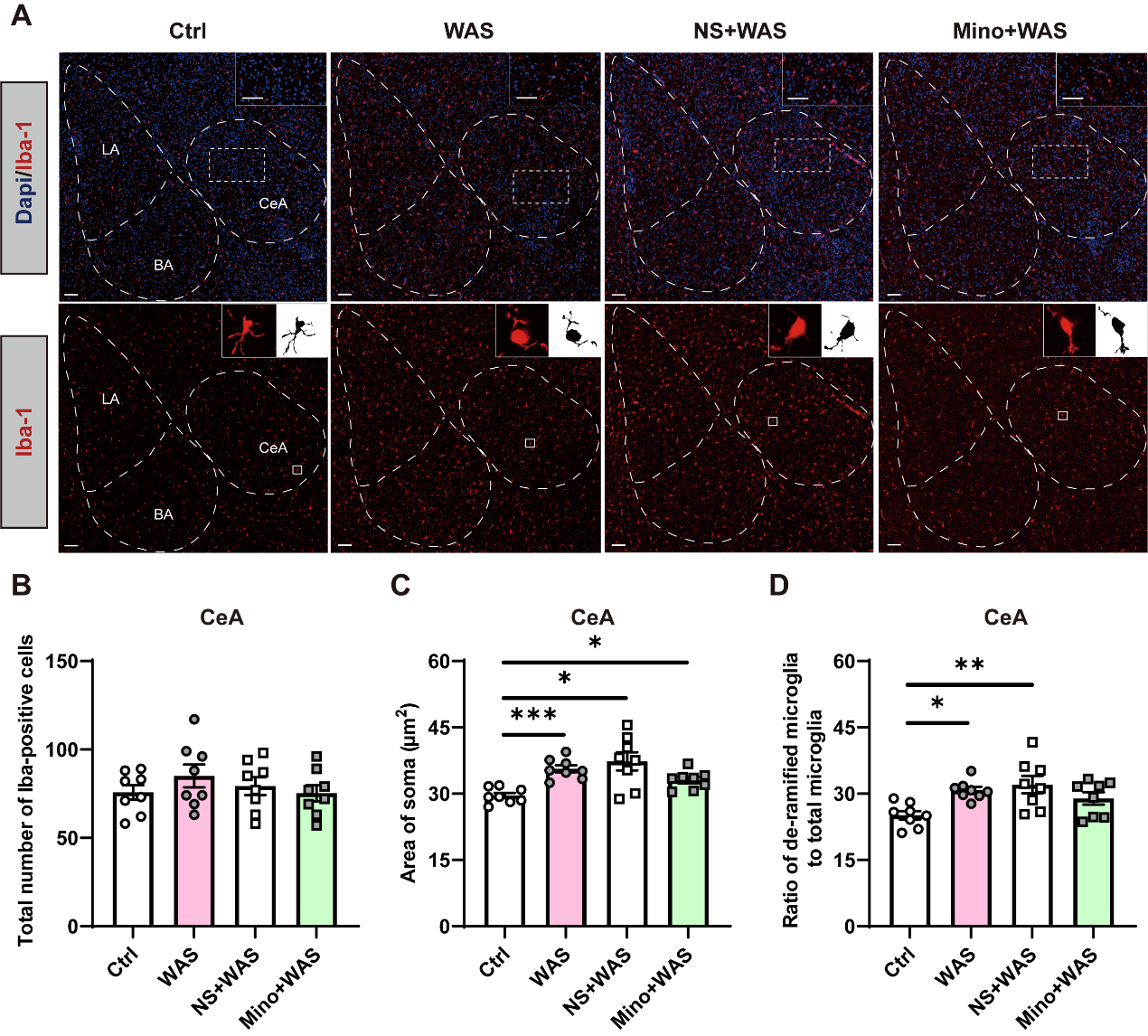
Figure S2**

**Figure S2** Minocycline infusions into the BLA have no significant influence on microglial activity in the CeA. **(A)** Representative images of Iba-1+ microglia immunofluorescent staining in the BLA and the CeA. A/P: -2.4 mm from bregma. Scale bar: 100 μm. **(B)** Number of Iba-1^+^ microglia in the CeA. n = 8 slices from 3-4 rats for each group, one-way ANOVA followed by Tukey's post hoc test, F _(3, 28)_ = 0.7677, p = 0.5218; Ctrl vs. WAS, p = 0.5855. **(C)** Quantification of the microglial soma area in the CeA. n = 8 slices from 3-4 rats for each group, one-way ANOVA with Welch’s correction, followed by Dunnett’s post-hoc test, p = 0.0002. **(D)** Comparison of the ratio of de-ramified microglia / total microglia in the CeA. All data are given as mean ± SEM. n = 8 slices from 3-4 rats for each group, one-way ANOVA followed by Tukey's post hoc test, F _(3, 28)_ =4.950, p = 0.0070. All data are given as mean ± SEM. *p < 0.05, **p < 0.01, ***p < 0.001. Ctrl: control, WAS: water avoidance stress, NS: normal saline, Mino: minocycline.

**
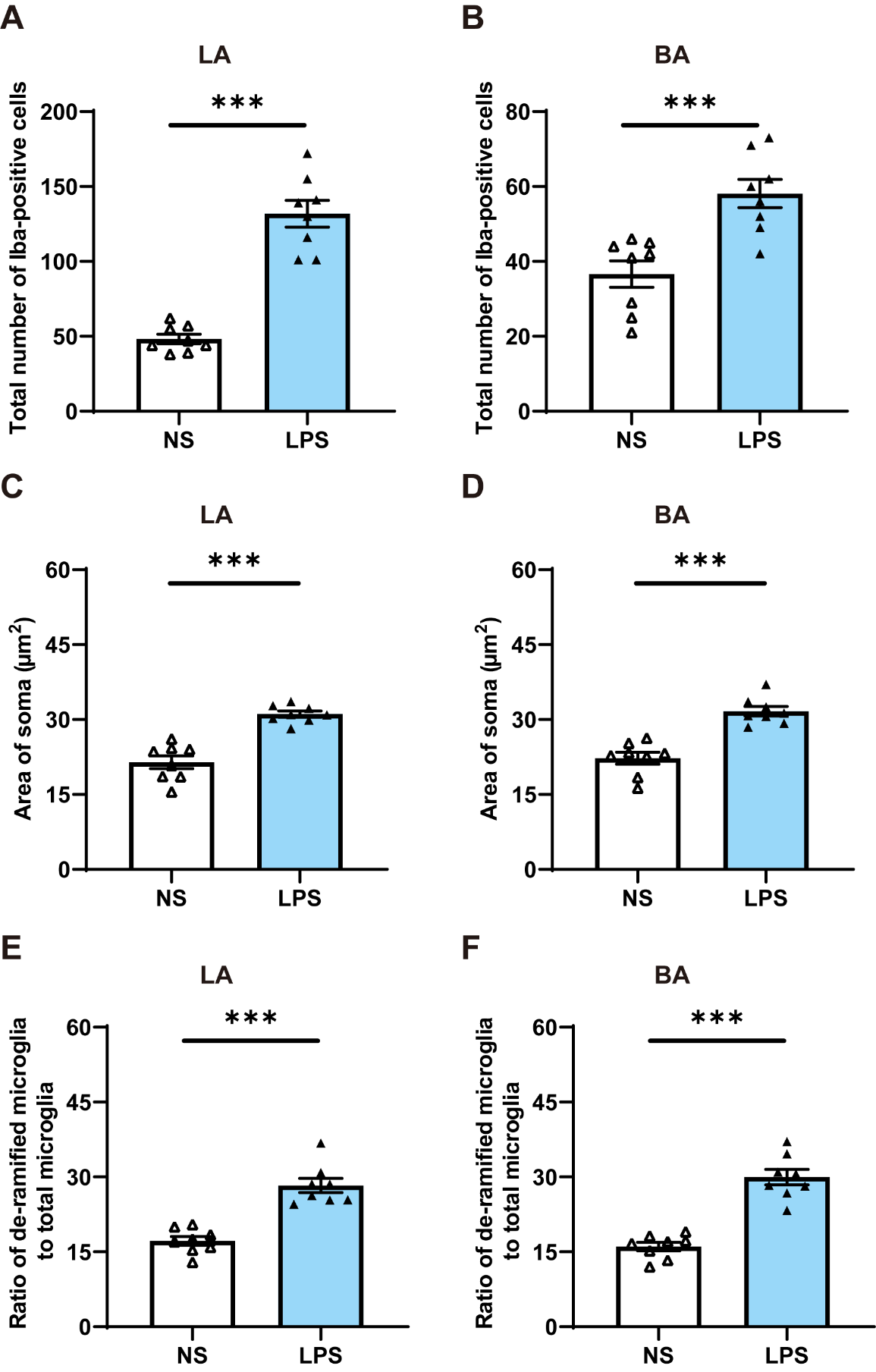
Figure S3**

**Figure S3** Lipopolysaccharide (LPS) treatment induces microglial activation in the lateral amygdala (LA) and the basal amygdala (BA). Number of Iba-1^+^ microglia in **(A)** LA (unpaired Student's t­test, p < 0.0001) and **(B)** BA (unpaired Student's t­test, p = 0.0009). Quantification of the microglial soma area in **(C)** LA (unpaired Student's t­test, p < 0.0001) and **(D)** BA (unpaired Student's t­test, p < 0.0001). Comparison of the ratio of de-ramified microglia / total microglia in **(E)** LA (unpaired Student's t­test, p < 0.0001) and **(F)** BA (unpaired Student's t­test, p < 0.0001). All data are given as mean ± SEM. n = 6 rats for each group. ***p < 0.001.

**Figure S4
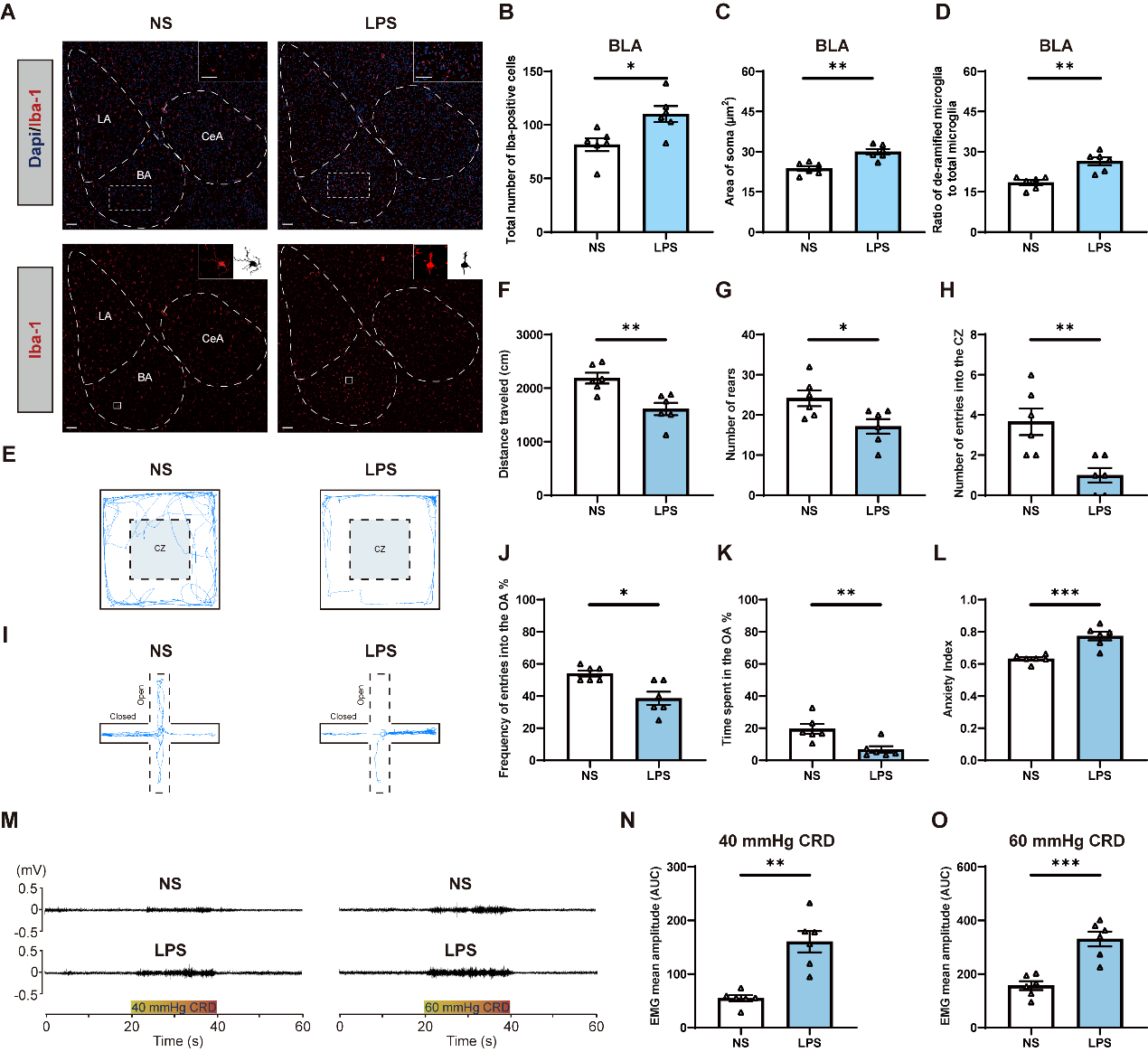
**

**Figure S4** Intra-BLA LPS treatment induces anxiety-like behaviors and visceral hypersensitivity in rats. **(A)** Representative images of Iba-1^+^ microglia immunofluorescent staining in the BLA 24-hours post local LPS or NS injection into the BLA. A/P: -2.4 mm from bregma. Scale bar: 100 μm. **(B)** Number of Iba-1^+^ microglia in the BLA. n = 6 slices from 3 rats for each group, unpaired Student’s t test, p = 0.0143. **(C)** Quantification of the microglial soma area in the BLA, unpaired Student’s t test, p = 0.0014. **(D)** Comparison of the ratio of de-ramified microglia / total microglia in the BLA, unpaired Student’s t test, p = 0.0011. **(E)** Representative activity traces in the open field test (OFT) 24-hours post intra-BLA LPS or NS treatment. The comparison of **(F)** total distance traveled (n = 6 rats for each group, unpaired Student’s t test, p = 0.0039), **(G)** total number of rears (unpaired Student’s t test, p = 0.0265), and **(H)** number of entries into the central zone (CZ) (unpaired Student’s t test, p = 0.0056) in the OFT. **(I)** Representative activity traces in the elevated plus maze test (EPMT). The comparison of **(J)** frequency of entries into the open arms (OA) (Mann-Whitney test, p = 0.0216), **(K)** time spent in the OA (Mann-Whitney test, p = 0.0087), and **(L)** the anxiety index (unpaired Student’s t test, p = 0.0006) in the EPMT. **(M)** Representative electromyogram recordings of visceromotor response (VMR) amplitude in response to 40 and 60 mmHg colorectal distension (CRD) 24-hours post intra-BLA LPS or NS treatment. Comparison of VMR amplitude in response to **(N)** 40 (unpaired Student’s t test, p = 0.0024) and **(O)** 60 (unpaired Student’s t test, p = 0.0003) mmHg CRD. All data are given as mean ± SEM. *p < 0.05, **p < 0.01, ***p < 0.001.

**Figure S5**

**
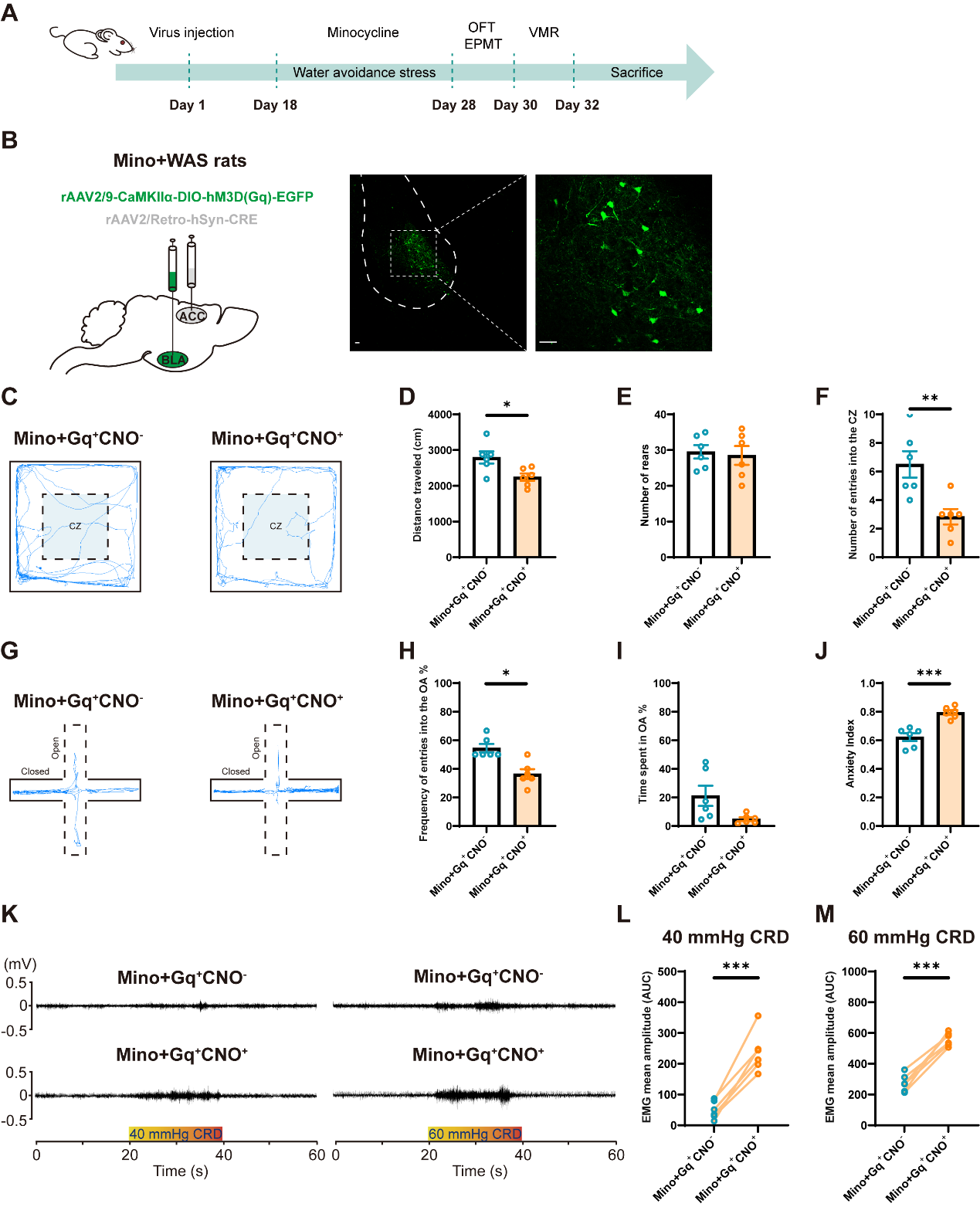
**

**Figure S5** Inhibition of BLA microglial activity in WAS rats followed by activation of the BLA-ACC glutamatergic pathway induces anxiety-like behaviors and visceral hypersensitivity. **(A)** A schematic diagram of the experimental design. **(B)** Graphical representation of CRE-virus microinjection in the ACC and DIO-Gq-virus microinjection in the BLA, and representative images of virus expression in the BLA. A/P: -2.4 mm from bregma. Scale bar: 100 μm. **(C)** Representative activity traces in the OFT. The comparison of **(D)** total distance traveled (unpaired Student’s t test, p = 0.0195), **(E)** total number of rears (unpaired Student’s t test, p = 0.7632), and **(F)** number of entries into the CZ (unpaired Student’s t test, p = 0.0065) in the OFT. **(G)** Representative activity traces in the EPMT. The comparison of **(H)** frequency of entries into the OA (Mann-Whitney test, p = 0.0108), **(I)** time spent in the OA (unpaired Student’s t test, p = 0.0701), and **(J)** the anxiety index in the EPMT (unpaired Student’s t test, p = 0.0003). **(K)** Representative EMG recordings of VMR amplitude in response to 40 and 60 mmHg CRD. Comparison of VMR amplitude in response to **(L)** 40 (paired Student’s t tests, p < 0.0001) and **(M)** 60 (paired Student’s t tests, p < 0.0001) mmHg CRD. n = 6 rats for each group. All data are given as mean ± SEM. *p < 0.05, **p < 0.01, ***p < 0.001.
